# Pro-inflammatory cytokine IFN-γ protects against renal fibrosis by promoting E3 ubiquitin ligase Trim21-mediated Loxl2 degradation in tubular epithelial cells

**DOI:** 10.1038/s41419-026-08850-7

**Published:** 2026-05-13

**Authors:** Hanlu Jiang, Mengru Gu, Mengzhu Tan, Xueling Chen, Xiaokai Yang, Yizhi Ren, Xiaoli Sun, Chunsun Dai

**Affiliations:** 1 Center for Kidney Diseases, the Second Affiliated Hospital of Nanjing Medical University, Nanjing, China; 2 Department of Clinical Genetics, the Second Affiliated Hospital of Nanjing Medical University, Nanjing, China

**Keywords:** Ubiquitylation, End-stage renal disease

## Abstract

The excessive accumulation of extracellular matrix (ECM) is a hallmark of renal interstitial fibrosis, its underlying mechanisms are incompletely understood. Here, we identify the E3 ubiquitin ligase Tripartite motif-containing protein 21 (Trim21) as a key regulator of this process. We found that Trim21 is upregulated in the tubular cells of fibrotic kidneys from both chronic kidney disease (CKD) patients and mouse models. Using tubular cell-specific Trim21 knockout mice, we demonstrated that Trim21 induction protects against ECM accumulation and renal fibrosis. Mechanistically, Trim21 binds to the N-terminal domain of Lysyl Oxidase-like 2 (Loxl2), promoting its ubiquitination and degradation, which in turn alleviates ECM deposition. Furthermore, we observed an upregulation of interferon-γ (IFN-γ) and its receptor in tubular cells during fibrosis. IFN-γ treatment increased Trim21 expression, reduced Loxl2 expression and renal fibrosis; critically, this protective effect was abolished in tubular-specific Trim21 knockout mice. In summary, our study defines a protective IFN-γ/Trim21/Loxl2 axis in the kidney, wherein IFN-γ signaling induces Trim21 to target Loxl2 for degradation, thereby mitigating fibrosis.

## Introduction

Chronic kidney disease (CKD) affects approximately 800 million individuals worldwide [1]. Its progression to end-stage renal disease leads to high morbidity, mortality, and significant healthcare costs [2, 3], making the prevention of CKD progression an urgent public health objective.

The central pathological driver of CKD is kidney fibrosis, characterized by the excessive deposition of extracellular matrix (ECM) proteins such as collagen [4]. While mesenchymal cells are a major source of ECM [5], numerous studies underscore the critical contribution for profibrotic tubular cells (e.g., Vcam1+ cells), which promote fibrosis through both direct ECM production and paracrine signaling [6]. This highlights tubular cells as a crucial focus for understanding kidney fibrogenesis.

In addition to ECM deposition, chronic inflammation is another hallmark of CKD, marked by the upregulation of cytokines like tumor necrosis factor-α and interleukin-6 [7]. Among these, interferon-γ (IFN-γ) is a pleiotropic cytokine with well-documented antiviral, antibacterial, and antitumor activities, as well as recognized antifibrotic properties across various tissues [8]. This antifibrotic potential has prompted the exploration of IFN-γ as a therapy for lung and liver fibrosis. However, its specific role and mechanistic actions in renal fibrosis remain ambiguous, with existing literature reporting conflicting results [9–13].

E3 ubiquitin ligases are pivotal regulators of cellular processes, and the Tripartite motif (Trim) family represents the largest such group in humans, with approximately 80 members [14, 15]. Characterized by a conserved structure, a RING domain for E2 binding, B-box domains, a coiled-coil region for dimerization, and a variable C-terminal substrate-binding domain [16–19], Trim proteins are increasingly implicated in renal fibrosis and specific members play divergent roles. For instance, Trim13, Trim27, Trim39 and Trim6 exacerbate injury in diabetic or lupus nephropathy [20–23], whereas Trim72 and Trim18 demonstrate protective effects against fibrosis [24, 25].

Ro52/Trim21 is recognized as an autoantigen in systemic lupus erythematosus and Sjögren’s syndrome [26]. Under baseline conditions, Trim2-null mice exhibit a normal phenotype. However, following tissue trauma such as ear tagging, they develop severe dermatitis that originates at the site of injury. These Trim21-deficient mice also display multiple systemic lupus-like features, including hypergammaglobulinemia, anti-DNA autoantibodies, proteinuria, and significant renal pathology [26, 27].

Beyond its well-established role in immunity [28, 29], emerging evidence has revealed context-dependent functions of TRIM21 in diverse kidney pathologies. In renal cell carcinoma (RCC), TRIM21 functions as a tumor suppressor by promoting ubiquitination-mediated degradation of oncogenic targets such as HIF-1α and SREBF1, thereby inhibiting tumor growth and metabolic reprogramming [30, 31].Additionally, studies have shown that Trim21 expression is upregulated in various renal injury models [32–34], and Trim21 global knockout alleviates kidney injury induced by ischemia‑reperfusion (IRI) and unilateral ureteral obstruction (UUO) [33, 34]. Our previous study demonstrated that Trim21 promotes Glut1 degradation, thereby modulates glycolysis in tubular cell [35], which was confirmed by Wen et al [36]. Despite these advances, the upstream regulatory mechanisms of TRIM21 and its specific function in tubular epithelial cells in coordinating inflammation and renal fibrosis remain to be elucidated.

Here, we identify a protective pathway in which IFN-γ signaling upregulates the E3 ubiquitin ligase Trim21 within tubular cells. We demonstrate that Trim21 targets the profibrotic protein Lysyl oxidase-like 2 (Loxl2) for ubiquitin-mediated degradation, thereby attenuating ECM accumulation. Our findings delineate a novel IFN-γ/Trim21/Loxl2 axis and propose Trim21 as a potential therapeutic target for curbing fibrosis in CKD.

## Results

### Upregulation of Trim21 in fibrotic kidneys of patients and mice with chronic kidney diseases

**Fig. 1:**
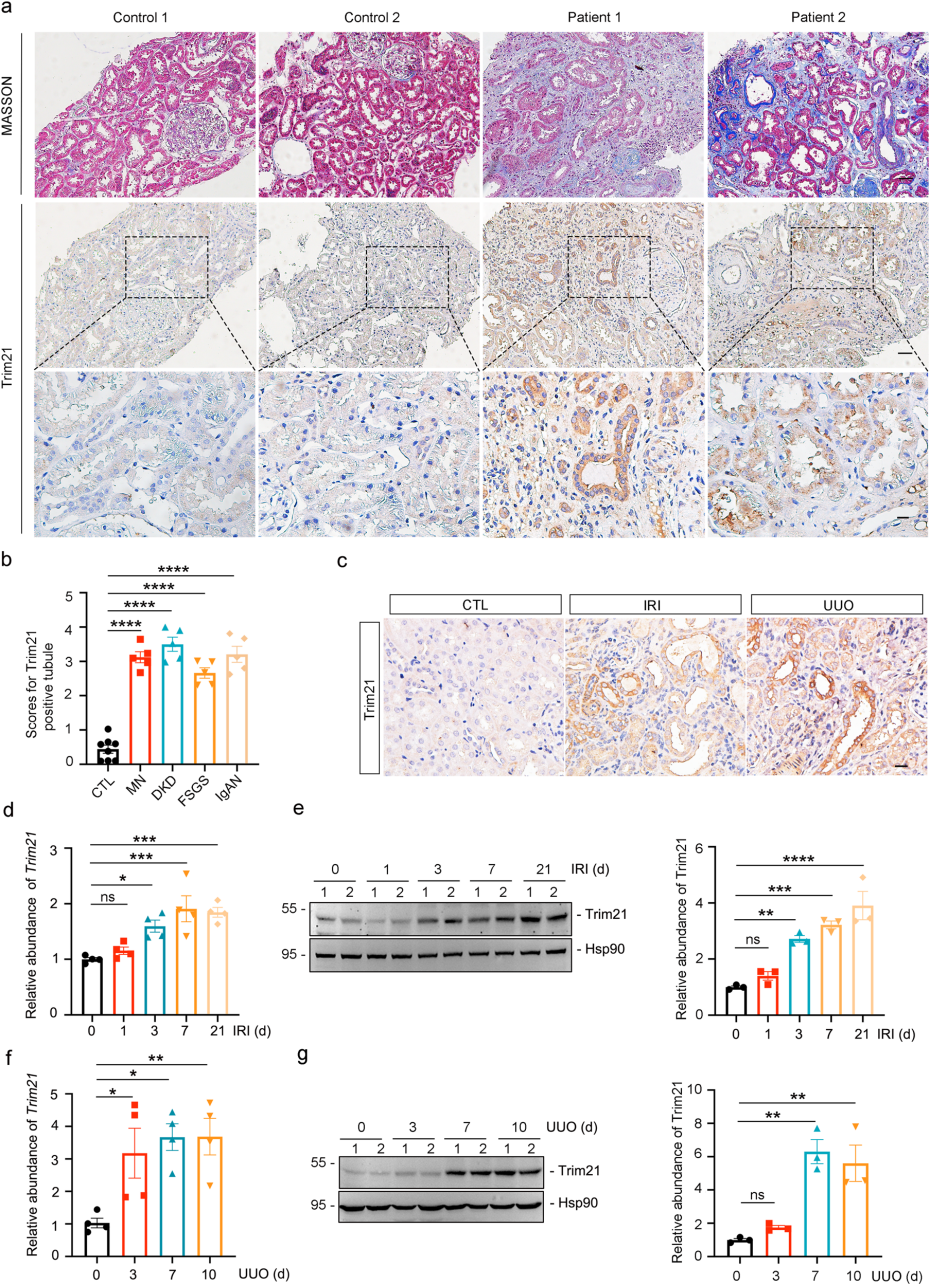
Trim21 expression is upregulated in fibrotic kidneys from patients with chronic kidney disease (CKD) and mouse models. **a** Representative Masson’s trichrome staining and Trim21 immunohistochemical staining in kidney sections from control subjects and patients with various CKD diagnoses: membranous nephropathy (MN), diabetic kidney disease (DKD), focal segmental glomerulosclerosis (FSGS), and IgA nephropathy (IgAN). Scale bars: 50 μm (overview), 20 μm (enlarged views). **b** Quantification of Trim21 staining intensity in human kidney sections (Control, *n* = 8; each CKD group, *n* = 5). **c** Immunohistochemical staining of Trim21 in kidney sections from sham, ischemia-reperfusion injury (IRI), and unilateral ureteral obstruction (UUO) mice. Scale bar: 20 μm. **d** Trim21 mRNA levels in mouse kidneys at indicated time points after IRI surgery (*n* = 4 per time point). **e** (Left) Representative western blot and (Right) quantitative analysis of Trim21 protein expression at different time points post-IRI (*n* = 3). (**f**) Trim21 mRNA levels in mouse kidneys at indicated time points after UUO surgery (*n* = 4 per time point). **g** (Left) Representative western blot and (Right) quantitative analysis of Trim21 protein expression at different time points post-UUO (*n* = 3). Data are presented as mean ± SEM. *p < 0.05, **p < 0.01, ***p < 0.001, ****p < 0.0001 versus control (day 0) by one-way ANOVA with Dunnett’s multiple comparisons test.

The Trim family, with around 80 members, is the largest group of E3 ubiquitin ligases in human. To investigate the role of Trim21 in renal fibrosis, we first enrolled adult CKD patients with diverse etiologies, such as membranous nephropathy (MN), diabetic kidney disease (DKD), focal segmental glomerulosclerosis (FSGS), and IgA nephropathy (IgAN), all of whom exhibited varying degrees of renal fibrosis (Supplementary Table 1). Immunohistochemical (IHC) analysis revealed minimal Trim21 expression in control kidneys but intense staining in fibrotic kidneys, particularly within dilated tubules (Fig. 1a, b). To validate these findings in an experimental setting, we established mouse models of renal fibrosis induced by ischemia-reperfusion injury (IRI) or unilateral ureteral obstruction (UUO). Mice were sacrificed at 0, 1, 3, 7, 14, and 21 days post-IRI, and at 0, 3, 7, and 10 days post-UUO. IHC staining demonstrated marked induction of Trim21 predominantly in tubular cells (Fig. 1c). Consistent with this, both qRT-PCR and Western blot analyses revealed that Trim21 expression began to increase at day 3 following either IRI or UUO surgery (Fig. 1d–g). Collectively, these results indicate that Trim21 is predominantly upregulated in tubular cells of fibrotic kidneys.

### Tubular cell-specific deletion of Trim21 exacerbates IRI or UUO -induced renal fibrosis in mice

**Fig. 2:**
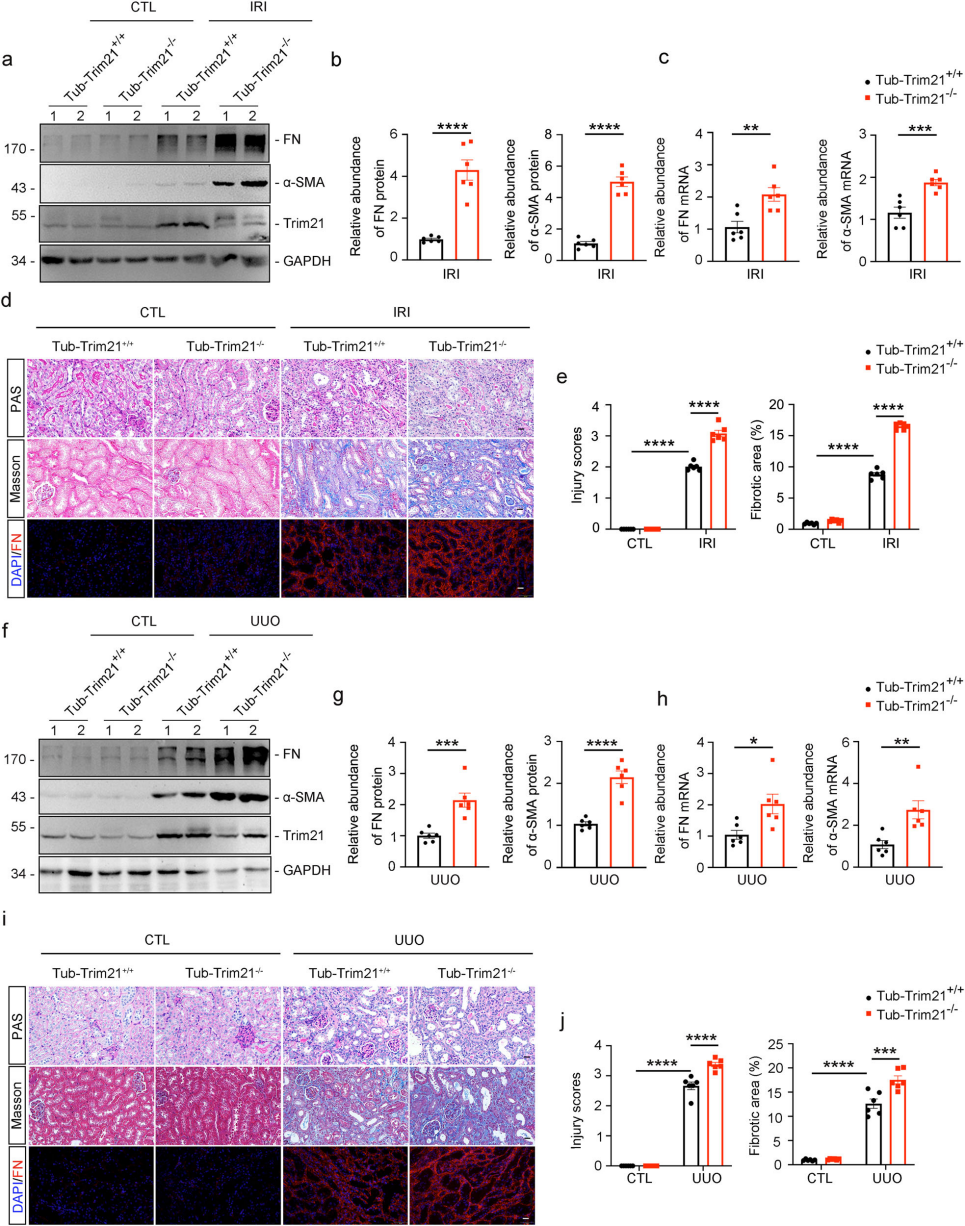
Tubular cell-specific deletion of Trim21 exacerbates renal fibrosis in IRI and UUO mouse models. **a–c** **a** Representative western blots and **b** quantitative analysis of fibronectin (FN) and α-SMA protein levels and **c** mRNA levels in kidneys from Tub-Trim21^+/+^ and Tub-Trim21^-/-^ mice under control (CTL) and ischemia-reperfusion injury (IRI) conditions (*n* = 6). **d** Representative kidney sections showing PAS staining, Masson’s trichrome staining, and FN immunofluorescence. **e** Quantification of tubular injury scores and fibrotic area (*n* = 6). Scale bar: 20 μm. **f–h** **f** Representative western blots and **g** quantitative analysis of FN and α-SMA levels and **h** mRNA levels in control and unilateral ureteral obstruction (UUO) kidneys (*n* = 6). **i**, **j** **i** Representative histology and immunofluorescence images and **j** their quantitative analysis from the UUO model (*n* = 6). Scale bar: 20 μm. Data are presented as mean ± SEM. *p < 0.05, **p < 0.01, ***p < 0.001, ****p < 0.0001; statistical significance was determined by two-tailed Student’s t-test (**b**, **c**, **g**, **h**) or two-way ANOVA with Tukey’s multiple comparisons test (**e**, **j**).

To directly investigate the functional role of Trim21 upregulation in tubular cells during renal fibrosis, we generated mice with tubular cell-specific knockout of Trim21 using the Cre-LoxP system (Supplementary Fig. 1a–d). These Tub-Trim21^-/-^ mice were born normal and exhibited comparable kidney histology and function to their Trim21^+/+^ littermates at 2 and 12 months of age (Supplementary Fig. 1e–k). Following kidney IRI surgery, Tub-Trim21^-/-^ mice showed a marked exacerbation of fibrosis on day 21, as evidenced by significantly increased production of FN and α-SMA, along with more severe tubular atrophy, compared to their Trim21^+/+^ littermates (Fig. 2a–e). A parallel exacerbation was observed in the UUO model, with Trim21-deficient mice displaying enhanced ECM deposition, tubular atrophy, and interstitial fibrosis on day 10 post-surgery (Fig. 2f–j). Collectively, these results demonstrate that tubular cells-specific deletion of Trim21 aggravates renal fibrosis induced by both IRI and UUO, indicating that Trim21 induction plays a protective role against the renal fibrosis.

To complement the genetic knockout approach, we also employed a knockdown strategy by injecting Trim21-specific short hairpin RNA (shRNA) via tail vein. Mice received two intravenous injections of Trim21-shRNA, at 5 days before and 3 days after UUO surgery, respectively (Supplementary Fig. 2a). This regimen effectively reduced Trim21 protein abundance in the kidneys (Supplementary Fig. 2b). Consistent with those observed in the knockouts, Trim21 knockdown significantly exacerbated UUO-induced renal fibrosis, leading to increased expression of FN and α-SMA, as well as more severe tubular atrophy (Supplementary Fig. 2c–f). These data from an independent loss-of-function model further confirm that Trim21 deficiency aggravates renal fibrosis.

### Trim21 mitigates extracellular matrix production and renal fibrosis through reducing Loxl2 abundance

**Fig. 3:**
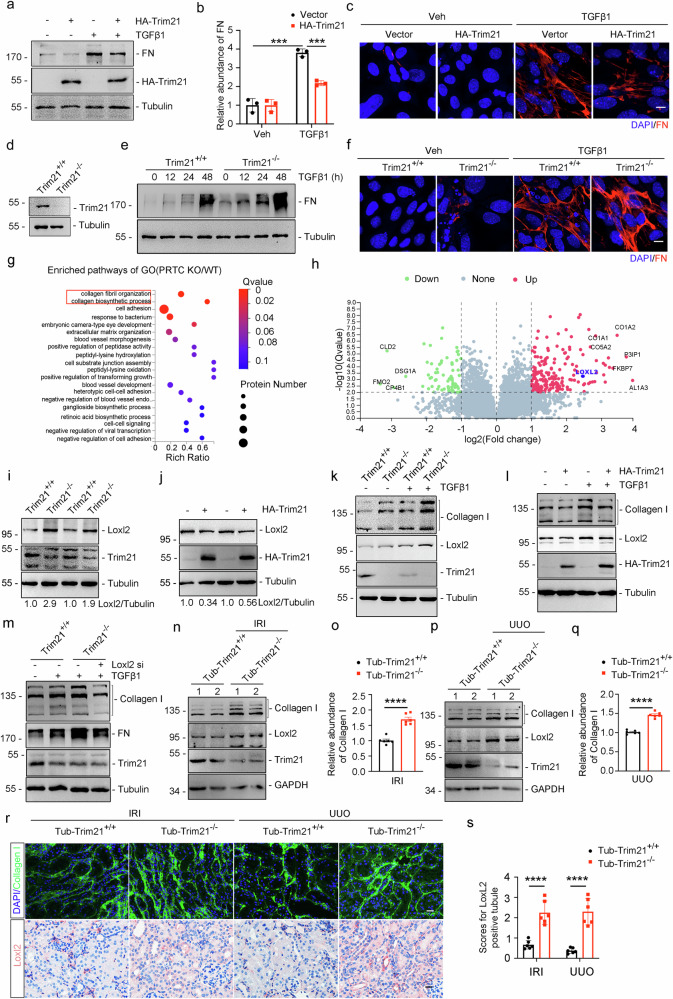
Loxl2 mediates the pro-fibrotic effect of Trim21 deficiency. **a–f** Trim21 negatively regulates ECM production: **a**, **b** Western blot and quantification show that HA-Trim21 overexpression suppresses TGF-β1-induced FN expression (*n* = 3). **c** FN immunofluorescence confirms this finding. **d** Western blot validates Trim21 ablation in primary TECs. **e** Time-course western blot and **f** immunofluorescence show that Trim21 knockout enhances TGF-β1-induced FN production. **g**, **h** Proteomic profiling: **g** GO biological process enrichment analysis and **h** volcano plot of differentially expressed proteins in Trim21-knockout versus control TECs. **i**, **j** Loxl2 is a key downstream target: Western blot analysis shows that **i** Trim21 knockout upregulates, while **j** overexpression downregulates Loxl2 protein levels. **k–m** Loxl2 is necessary for the pro-fibrotic phenotype: Western blots show that **k** Trim21 knockout upregulates Collagen I and Loxl2, **l** Trim21 overexpression downregulates them, and **m** silencing Loxl2 rescues the enhanced Collagen I and FN expression in Trim21-knockout TECs. **n–s** Validation in mouse models: **n**, **o** IRI and **p**, **q** UUO mouse kidneys show increased Collagen I and Loxl2 in Tub-Trim21^-/-^ mice. **r** Representative IHC staining and **s** quantification confirm elevated Loxl2 in fibrotic kidneys of knockout mice (*n* = 6). Data are mean ± SEM. ***p < 0.001, ****p < 0.0001 by two-tailed Student’s t-test. Scale bar: 5μm (c, f), Scale bar: 20 μm (**r**).

To elucidate the mechanisms by which Trim21 protects against renal fibrosis, we treated cultured tubular cells with TGF-β1, a well-established pro-fibrotic cytokine [37]. Ectopic overexpression of Trim21 significantly attenuated TGF-β1-induced FN production (Fig. 3a–c). Conversely, genetic knockout of Trim21 exacerbated the TGF-β1-mediated upregulation of FN (Fig. 3d–f). These data demonstrate that Trim21 may suppress TGF-β1-induced extracellular matrix production in tubular cells.

To determine whether Trim21 attenuates extracellular matrix production by modulating the canonical Smad signaling pathway, we examined Smad1/2/3/5 phosphorylation and Smad3 nuclear translocation in cultured tubular cells. As expected, TGF-β1 stimulation markedly induced the phosphorylation of Smad signaling components and promoted Smad3 nuclear accumulation (Supplementary Fig. 3a–d). However, neither overexpression nor ablation of Trim21 significantly altered these processes (Supplementary Fig. 3a–d). These results indicate that the anti-fibrotic effect of Trim21 is independent of the canonical Smad pathway.

To elucidate the mechanism by which Trim21 regulates ECM production, we conducted a proteomic analysis comparing Trim21^+/+^ and Trim21^-/-^ tubular cells. Gene Ontology (GO) term analysis of the differentially expressed proteins revealed significant enrichment in pathways related to collagen fibril organization, collagen biosynthetic process, and cell adhesion in Trim21^-/-^ cells (Fig. 3g). Notably, among the upregulated proteins, lysyl oxidase-like 2 (Loxl2), a key enzyme promoting collagen cross-linking and tissue stiffness, was markedly elevated in Trim21^-/-^ cells (Fig. 3h), identifying it as a potential downstream effector.

Having identified Loxl2 from the proteomic screen, we confirmed its inverse relationship with Trim21. Western blot analysis verified that Loxl2 protein was induced in Trim21^*-/-*^ cells, whereas Trim21 overexpression reduced its abundance (Fig. 3i, j). Functionally, knockout of Trim21 enhanced TGF-β1-induced collagen deposition, an effect that was reversed by Trim21 overexpression (Fig. 3k, l). Most importantly, knockdown of Loxl2 abolished the excessive extracellular matrix production triggered by TGF-β1 in Trim21^*-/-*^ cells (Fig. 3m, Supplementary Fig. 4a, b), establishing Loxl2 as a critical downstream effector. This regulatory axis was consistently observed in vivo, as Trim21 knockout led to increased Loxl2 and collagen levels in both IRI- and UUO-induced fibrotic kidneys (Fig. 3n–s). Collectively, these data demonstrate that Trim21 modulates extracellular matrix accumulation and renal fibrosis by negatively regulating Loxl2.

### Trim21 promotes the degradation of Loxl2 via the ubiquitin-proteasome pathway

**Fig. 4:**
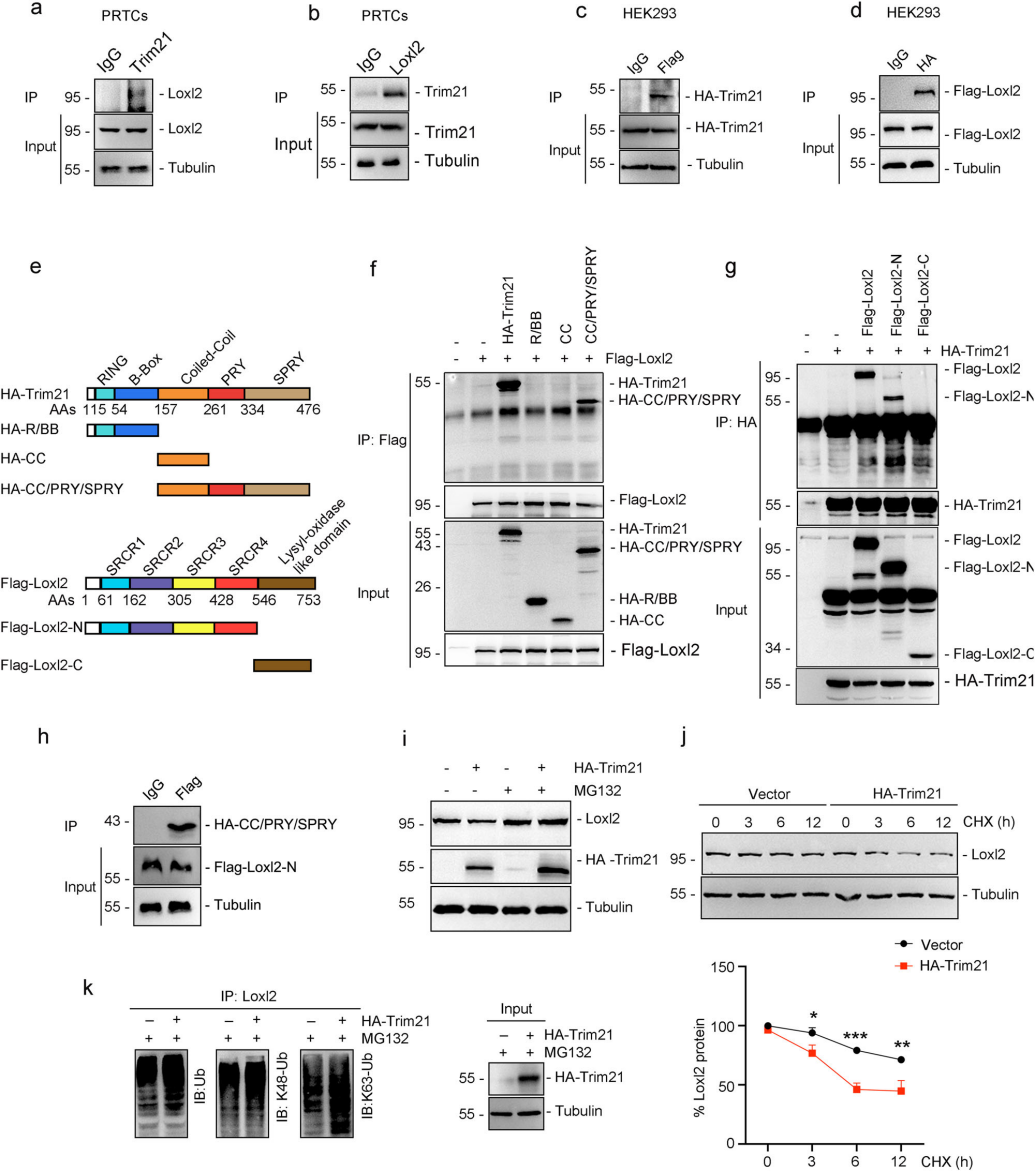
Trim21 targets Loxl2 for ubiquitin-mediated degradation. **a–d** Trim21 interacts with Loxl2: Endogenous co-immunoprecipitation (Co-IP) in primary TECs using **a** Trim21 or **b** Loxl2 antibody. Exogenous Co-IP in HEK293 cells using **c** Flag or **d** HA antibody. **e–g** Mapping the interaction domains: **e** Schematics of Trim21 and Loxl2 wild-type and deletion mutants. **f** The PRY-SPRY domain of Trim21 and **g** the N-terminal domain of Loxl2 are required for their interaction. **h** Direct domain interaction confirmed by Co-IP between the HA-tagged Trim21 PRY-SPRY domain and the Flag-tagged Loxl2 N-terminal domain in HEK293 cells. **i** Proteasome inhibition by MG132 (5 μM, 6 h) blocks Trim21-mediated Loxl2 downregulation. **j** Cycloheximide (CHX, 50 μg/mL) chase assay shows that Trim21 overexpression shortens the half-life of endogenous Loxl2 protein in primary TECs (*n* = 3). **k** In vivo ubiquitination assay in primary TECs demonstrates that Trim21 overexpression promotes polyubiquitination of Loxl2. Data are presented as mean ± SEM. *p < 0.05, **p < 0.01, ***p < 0.001 by two-tailed Student’s t-test.

Given its known function as an E3 ubiquitin ligase, we hypothesized that Trim21 promotes Loxl2 degradation through direct targeting. We first ruled out transcriptional regulation, as *Loxl2* mRNA levels were comparable in Trim21^*+/+*^ and Trim21^*-/-*^ tubular cells (Supplementary Fig. 5a–e). To investigate whether there is a direct interaction between Trim21 and Loxl2, we performed co‑immunoprecipitation (Co‑IP) assays. As shown in Fig. 4a, when lysates were immunoprecipitated with a specific antibody against Trim21, subsequent immunoblotting clearly detected Loxl2. Reciprocally, immunoprecipitation using an antibody against Loxl2 also pulled down Trim21 (Fig. 4b). In contrast, no specific bands were observed when control IgG was used for immunoprecipitation. Co-IP assays in HEK293 cells co-expressing HA-tagged Trim21 and Flag-tagged Loxl2 also confirmed their physical interaction and complex formation (Fig. 4c, d). Loxl2 have a highly conserved carboxyl terminus consisting of a copper-ion-binding site, the lysyl tyrosine quinone (LTQ), and a cytokine receptor-like (CRL) structural domain [38, 39]. To map the interaction domains, we expressed full-length or truncated versions of both proteins in HEK293 cells. This analysis revealed that the PRY/SPRY domain of Trim21 mediates binding to the N-terminal region of Loxl2 (Fig. 4e–h).

To verify whether Trim21 targets Loxl2 for proteasomal degradation, we first inhibited the proteasome with MG132. Treatment with MG132 increased basal Loxl2 levels and effectively blocked the reduction in Loxl2 induced by Trim21 overexpression (Fig. 4i). We next assessed the direct impact of Trim21 on Loxl2 protein stability by measuring its half-life after inhibiting new protein synthesis with cycloheximide (CHX). The half-life of Loxl2 was largely shortened in cells overexpressing HA-Trim21 compared to controls (Fig. 4j).

To characterize the type of ubiquitin linkage on Loxl2 catalyzed by Trim21, we co-expressed HA-Trim21 and Flag-Loxl2 in HEK293 cells and performed immunoprecipitation. The results showed that overexpression of Trim21 significantly increased the levels of both Lys48-linked and Lys63-linked polyubiquitin chains on Loxl2 compared to the control (Fig. 4k). These findings indicate that Trim21 promotes the conjugation of both K48- and K63-linked ubiquitin chains to Loxl2, which facilitates its degradation. Taken together, these results demonstrate that Trim21 binds to Loxl2 and promotes its ubiquitination and proteasomal degradation.

### IFN-γ alleviates renal fibrosis by upregulating Trim21 expression

**Fig. 5:**
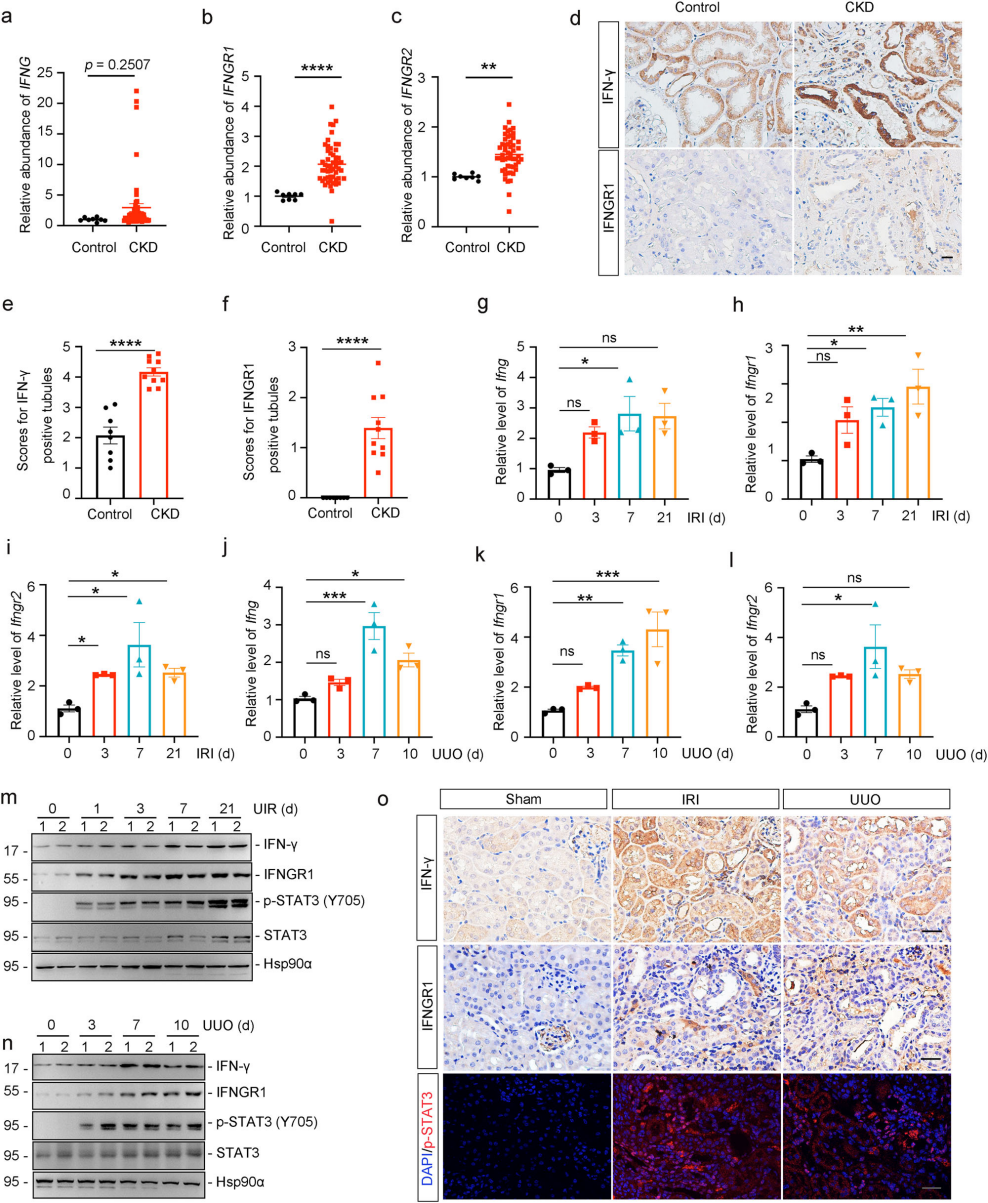
The interferon-γ signaling pathway is activated in tubular cells of fibrotic kidneys. **a–c** Analysis of a human CKD dataset (GSE66494) shows mRNA levels of **a** IFNG, **b** IFNGR1, and **c** IFNGR2. **d–f** Protein expression in human CKD kidneys: **d** Representative immunohistochemical staining for IFN-γ and IFNGR1. Scale bar: 20 μm. **e**, **f** Semi-quantitative analysis of **e** IFN-γ and **f** IFNGR1 staining intensity (*n* = 10). **g–i** mRNA levels of **g** Ifng, **h** Ifngr1, and **i** Ifngr2 in mouse kidneys after ischemia-reperfusion injury (IRI). **j–l** mRNA levels of **j** Ifng, **k** Ifngr1, and **l** Ifngr2 in mouse kidneys after unilateral ureteral obstruction (UUO). **m**, **n** Western blot analysis of IFN-γ, IFNGR1, and phosphorylated STAT3 (p-STAT3) protein levels in **m** IRI and **n** UUO mouse kidneys. **o** Representative immunohistochemical images of IFN-γ, IFNGR1, and p-STAT3 in kidneys from IRI and UUO mice. Scale bar: 20 μm. Data are presented as mean ± SEM. *p < 0.05, **p < 0.01, ***p < 0.001, ****p < 0.0001 by two-tailed Student’s t-test (**a–c**, **e**, **f**) or one-way ANOVA with Dunnett’s multiple comparisons test (**g–l**).

IFN-γ is a pleiotropic cytokine known for its antiviral, antibacterial, and antitumor activities, as well as its recognized antifibrotic effects across various tissues [8]. Owing to this potential, IFN-γ has been explored as a therapeutic agent for lung and liver fibrosis. However, its specific role in renal fibrosis remains less clear, with existing studies reporting inconsistent results [9–13]. Microarray analysis of CKD patient kidneys showed a non-significant trend for increased *IFNG* mRNA (Fig. 5a), but significant elevations in *IFNGR1* and *IFNGR2* mRNA levels (Fig. 5b, c). Immunohistochemistry of human renal biopsies from CKD patients revealed significantly higher IFN-γ expression in tubules, but not glomeruli, compared to control tissues (Fig. 5d, e). Interestingly, its receptor, IFNGR1, was specifically upregulated in renal tubules (Fig. 5d, f). These clinical findings were corroborated in mouse fibrotic kidneys (IRI and UUO), which exhibited increased renal mRNA expression of *Ifng*, *Ifngr1*, and *Ifngr2* (Fig. 5g–l). At the protein level, immunoblotting confirmed upregulation of IFN-γ, IFNGR1, and phosphorylated STAT3 (p-STAT3) in fibrotic mouse kidneys (Fig. 5m, n). IHC and immunofluorescence staining further localized this response to injured tubules (TECs), showing increased IFN-γ/IFNGR1 expression and enhanced STAT3 phosphorylation with nuclear translocation (Fig. 5o). Collectively, these data demonstrate activation of the IFN-γ–IFNGR–STAT3 signaling axis specifically within injured tubules during renal fibrosis.

**Fig. 6:**
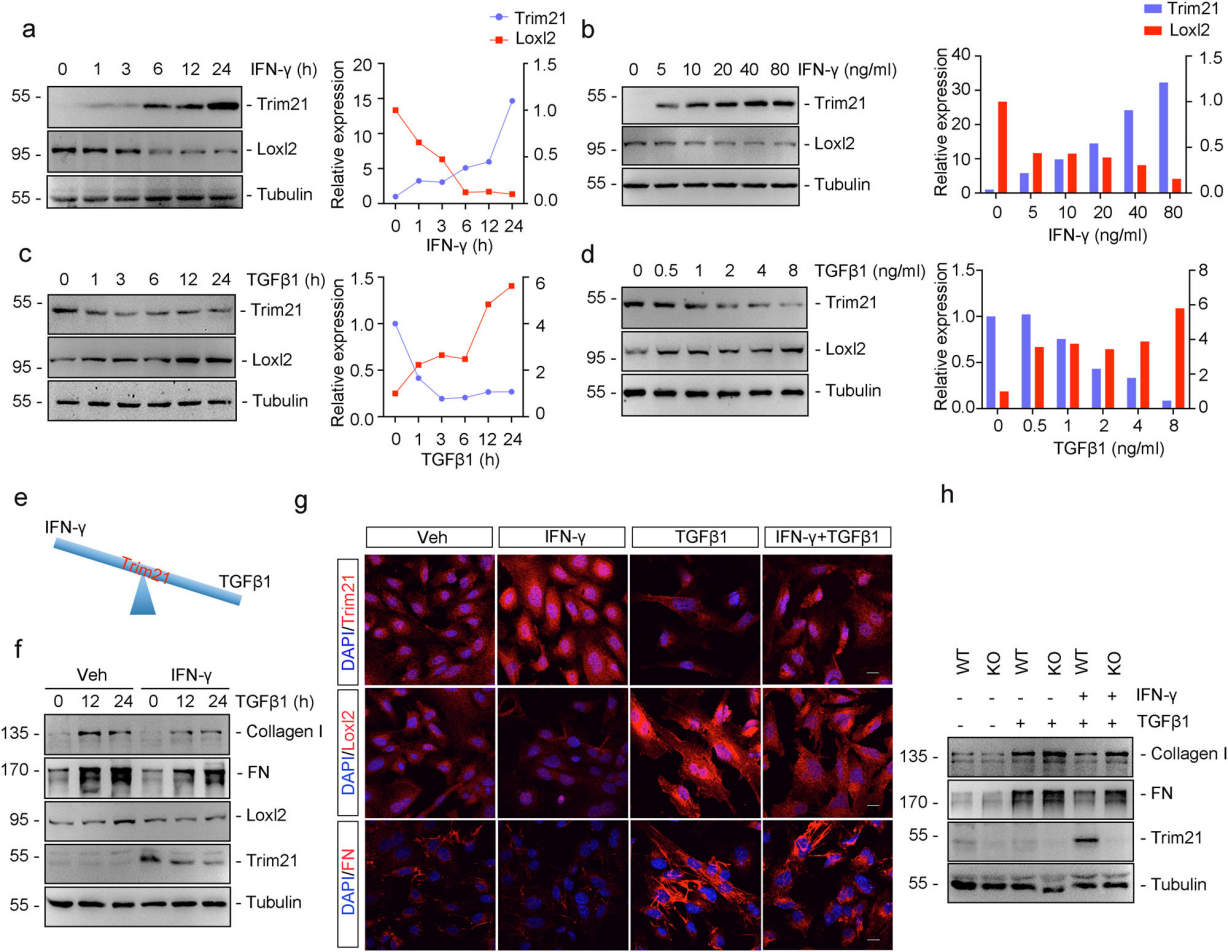
IFN-γ ameliorates TGF-β1-induced extracellular matrix production in tubular by upregulating Trim21. **a**, **b** IFN-γ upregulates Trim21 and downregulates Loxl2 in a **a** time- and **b** dose-dependent manner in primary TECs. **c**, **d** TGF-β1 downregulates Trim21 and upregulates Loxl2 in a **c** time- and **d** dose-dependent manner. **e** Schematic diagram illustrating the opposing regulation of Trim21 by IFN-γ and TGF-β1. **f** Western blot analysis shows that pretreatment with IFN-γ attenuates TGF-β1-induced upregulation of Loxl2 and the ECM proteins FN and Collagen I. **g** Immunofluorescence staining of Trim21 (red), Loxl2 (red), and FN (red) in TECs, confirming the findings in (**f**). Scale bar: 5 μm. **h** The protective effect of IFN-γ is Trim21-dependent: IFN-γ fails to suppress TGF-β1-induced Loxl2 and ECM protein production in Trim21-knockout (KO) TECs.

Given that interferon signaling can induce Trim21 expression [40], we investigated whether IFN-γ specifically upregulates Trim21 in tubular cells. Treatment of primary tubular cells with recombinant IFN-γ resulted in a time- and dose-dependent increase in Trim21 protein levels, which was accompanied by a concomitant decrease in Loxl2 (Fig. 6a, b). In contrast, the pro-fibrotic cytokine TGF-β1 downregulated Trim21 expression (Fig. 6c, d), revealing an antagonistic relationship between IFN-γ and TGF-β1 in regulating Trim21 within the renal microenvironment (Fig. 6e). Functionally, IFN-γ treatment attenuated TGF-β1-induced extracellular matrix production in wild-type tubular cells (Fig. 6f, g). Crucially, this protective effect of IFN-γ was completely abolished in Trim21-knockout tubular cells (Fig. 6h), demonstrating that Trim21 is essential for the anti-fibrotic action of IFN-γ.

To assess the effect of IFN-γ in vivo, we administered an IFN-γ expression plasmid to mice via tail vein injection (Supplementary Fig. 6a). qRT-PCR confirmed successful gene delivery in the liver (Supplementary Fig. 6b, c). Western blot analysis confirmed increased protein levels of both IFN-γ and its downstream target Trim21 in both of the liver and kidney (Supplementary Fig. 6d, e). Immunohistochemistry results showed strong IFN-γ immunoreactivity along the luminal surface of renal tubules, compared to a more restricted expression pattern in hepatocytes (Supplementary Fig. 6f).

**Fig. 7:**
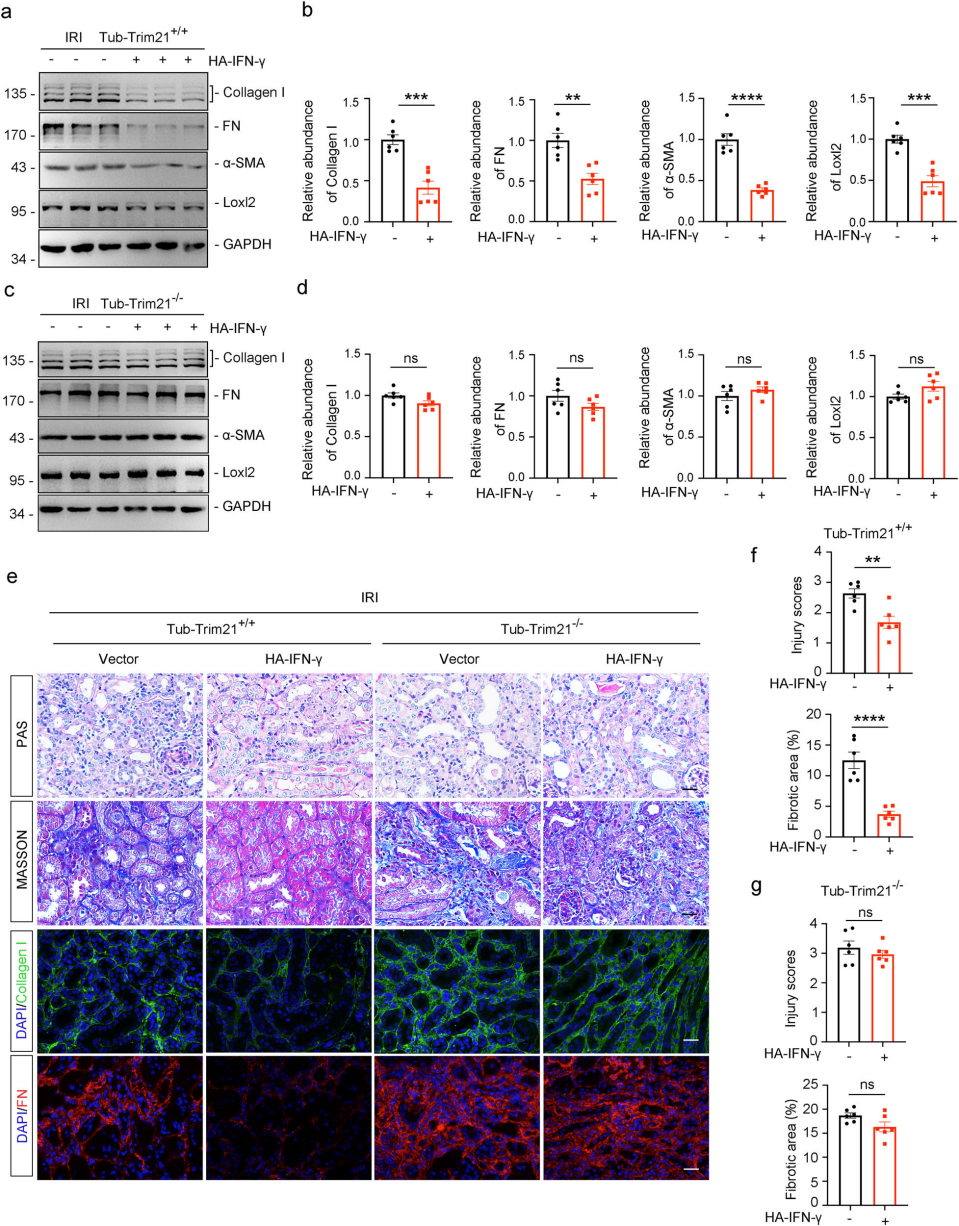
IFN-γ ameliorates renal fibrosis primarily through induction of Trim21 in vivo. **a**, **b** IFN-γ plasmid injection reduces fibrosis in wild-type mice: **a** Western blot analysis shows decreased levels of Loxl2, Collagen I, FN and α-SMA. **b** Semi-quantitative analysis of the protein levels (*n* = 6). **c**, **d** IFN-γ loses its anti-fibrotic effect in Trim21 knockout mice: **c** Western blot analysis demonstrates that IFN-γ plasmid injection fails to reduce Loxl2, Collagen I, FN or α-SMA in Trim21^-/-^ kidneys. **d** Semi-quantitative analysis of the protein levels (*n* = 6). **e** Histological analysis: Representative images of PAS staining, Masson’s trichrome staining and immunohistochemical staining for Collagen I and FN in kidney sections from Trim21^+/+^ and Trim21^-/-^ mice with or without IFN-γ plasmid treatment. Scale bar: 20 μm. **f**, **g** Quantification of (**f**) tubular injury scores and fibrotic area in Trim21^+/+^ kidneys, and (**g**) in Trim21^-/-^ kidneys, after IRI with or without IFN-γ treatment (*n* = 6). Data are presented as mean ± SEM. *p < 0.05, **p < 0.01, ***p < 0.001 by two-tailed Student’s t-test.

To evaluate the therapeutic potential of IFN-γ, we administered an IFN-γ plasmid on days 10 and 17 following IRI surgery. In wild-type mice, IFN-γ treatment significantly reduced the expression of Loxl2 in renal tissue, concomitant with a marked attenuation of extracellular matrix accumulation and structural damage (Fig. 7a, b, e, f). This protective effect was largely abolished in Trim21^*-/-*^ mice, with IFN-γ treatment showing minimal impact on Loxl2 levels or fibrosis (Fig. 7c-e, g). These results indicate that the anti-fibrotic effects of IFN-γ in kidneys are primarily mediated through Trim21.

## Discussion

**Fig. 8:**
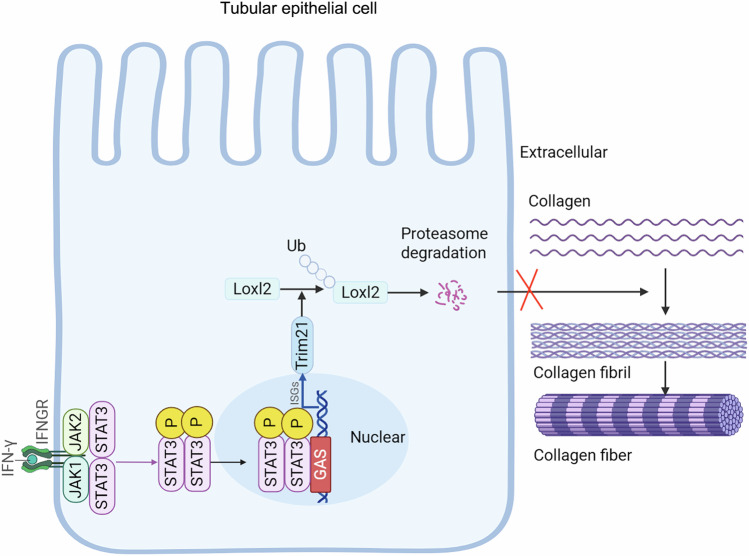
Schematic model of the IFN-γ/Trim21/Loxl2 axis in renal fibrosis. Elevated IFN-γ binds to its receptor (IFNGR), leading to STAT3 phosphorylation, nuclear translocation, and transcriptional upregulation of Trim21. Subsequently, Trim21 binds to Loxl2, promoting its ubiquitination and proteasomal degradation, thereby inhibiting extracellular matrix accumulation and fibrosis. Conversely, Trim21 deficiency stabilizes Loxl2, leading to its accumulation and exacerbation of renal fibrosis. This figure was created with BioRender. https://BioRender.com/p6dzxet.

This study shows that Trim21 expression is upregulated in fibrotic kidneys. Functionally, knockout of Trim21 in tubular cells significantly attenuates renal fibrosis. Mechanistically, IFN-γ upregulate Trim21 expression which interacts with and ubiquitinates the pro-fibrotic protein Loxl2, targeting it for proteasomal degradation. Thus, our findings establish a novel IFN-γ/Trim21/Loxl2 pathway and position Trim21 as a potential therapeutic target for chronic kidney diseases (Fig. 8).

Patients with CKD and cardiovascular disease exhibit a state of chronic inflammation, characterized by the induction of cytokines like tumor necrosis factor-α and interleukin-6 [7]. Among these, IFN-γ has demonstrated potent anti-fibrotic effects. In a rat subtotal nephrectomy (SNx) model, administration of 400 units/day inhibited myofibroblast activation, reduced renal fibrosis, and preserved renal function [12]. The mechanism of IFN-γ signaling involves Trim21, which is induced by interferon in macrophages and promotes the ubiquitination and transcriptional activity of interferon regulatory factor 8 (IRF8), thereby enhancing pro-inflammatory cytokine production and host defense [41]. In this study, we found that the IFN-γ pathway is highly active in tubular cells of fibrotic kidneys, as indicated by the upregulation of IFN-γ, its receptors, and its downstream effector, phosphorylated Stat3. We further demonstrated that IFN-γ reduces extracellular matrix production in tubular cells by transcriptionally upregulating Trim21 expression, a classic interferon-induced protein [42, 43]. Supporting a protective role, tubule-specific knockout of Trim21 resulted in more severe fibrosis following injury. This indicates that the upregulation of Trim21 in tubular cells represents a compensatory anti-fibrotic mechanism in the diseased kidney.

The pro-fibrotic role of Trim21 reported in global knockout models appears to contradict our findings. The same group initially reported that Trim21 exacerbates acute kidney injury by promoting GPX4 ubiquitination and ferroptosis [33]. They subsequently demonstrated that global Trim21 knockout alleviates renal fibrosis through enhanced autophagic degradation of mature TGF-β1 [34]. In contrast, our study shows that specific knockout of Trim21 in renal tubular epithelial cells markedly aggravates renal fibrosis in both IRI and unilateral UUO models—consistent with observations by Wen et al.

This apparent discrepancy is likely explained by the fundamental difference in genetic targeting strategies. Global knockout models lack Trim21 in all cell types, including infiltrating macrophages, fibroblasts, and lymphocytes—key drivers of fibrosis that may rely on Trim21 to promote TGF-β1 maturation or pro-inflammatory signaling. The beneficial effect of global deletion may therefore stem from modulating the microenvironment. In contrast, our conditional model uncovers the intrinsic role of Trim21 in TECs, where it appears to be required for stress adaptation and organelle homeostasis. The loss of this intrinsic protective mechanism in tubules may overwhelm any potential anti-inflammatory benefits derived from systemic knockout. Together, these results highlight the cell type‑dependent functional diversity of Trim21.

In the kidney, several Trim21 substrates have been identified, including Hif1a, GPX4, mature TGF-β1, GLUT1, PFKP, and FoxO3a [30, 33–36, 44]. In this study, we add Loxl2 to this list. Although we previously showed Trim21 inhibits glycolysis by degrading GLUT1 in tubular cells, its direct role in ECM regulation remained unclear. Here, we demonstrate that Trim21 attenuates ECM accumulation by targeting LOXL2. This multi-targeting capacity enables Trim21 to coordinate protective responses against diverse pathological insults. Loxl2 is a well-characterized extracellular enzyme that promotes ECM remodeling and fibrosis in various tissues [45]. Previous studies reported that a Loxl2 inhibitor ameliorates fibrosis by suppressing the TGF-β1-mediated p38 MAPK pathway [46], while Yang et al. demonstrated that Loxl2 cooperates with TGF-β1 via the PI3K/AKT/mTORC1 axis to promote myofibroblast transformation and migration [47]. It has been reported that Loxl2 can enter the nucleus to play the function of deacetylation, participate in histone modification, and affect tumor proliferation and metastasis [48]. In addition, Loxl2 directly binds to Snail1 in the nucleus to affect its nuclear localization and protein stability, and these functions do not depend on its amine oxidase action [49]. Therefore, our finding that Trim21 targets Loxl2 for degradation reveals a dual anti-fibrotic mechanism. First, by reducing Loxl2 secretion, Trim21 limits its direct extracellular action in collagen cross-linking, which impedes collagen degradation. Second, by depleting intracellular Loxl2, Trim21 disrupts key pro-fibrotic signaling pathways, ultimately mitigating renal interstitial fibrosis.

Ubiquitination is a versatile post-translational modification where specific chain linkages, such as K48 and K63, confer distinct functional outcomes [50, 51]. E3 ubiquitin ligases are central to this process, determining substrate specificity by transferring ubiquitin from E2 conjugating enzymes to target proteins. Trim44 was identified as an E3 ligase for Loxl2 in gastric cancer cells [52]. However, its role in kidney disease appears contradictory. Single-cell RNA sequencing data from patients with diabetic kidney disease (DKD) show high expression of Trim44 in injured proximal tubular cells [53], and animal studies indicate that Trim44 exacerbates cardiac hypertrophy and fibrosis [54, 55]. Given that Loxl2 is a known promoter of fibrosis, these findings suggest that Trim44 is unlikely to function as a degrader of Loxl2 in fibrotic diseases, as its own expression correlates with a pro-fibrotic phenotype. In contrast, our study demonstrated that Trim21 promotes both K48- and K63-linked polyubiquitination of Loxl2. While K48-linked chains typically target proteins for proteasomal degradation, providing a clear mechanism for the observed reduction in Loxl2, the role of K63-linked ubiquitination is more enigmatic. K63 linkages are generally associated with non-proteolytic functions, including signal transduction and protein trafficking. Therefore, the functional consequence of the K63-linked ubiquitination of Loxl2 by Trim21 remains an important and intriguing question for future investigation.

In summary, we demonstrate that the induction of Trim21 by IFN-γ attenuates renal fibrosis by inhibiting Loxl2-dependent ECM accumulation. Given the central role of Trim21 as an E3 ubiquitin ligase in this protective pathway, our findings suggest that using its activity, for instance, through emerging technologies like PROTACs [56], could represent a promising therapeutic strategy for CKD.

## Materials and Methods

### Human renal biopsy samples

Human kidney biopsy specimens were obtained from the Center for Kidney Diseases at the Second Affiliated Hospital of Nanjing Medical University. The adjacent healthy kidney tissues from patients with kidney tumors from the Second Affiliated Hospital of Nanjing Medical University were used as controls (*n* = 8). The renal fibrosis group (*n* = 20) was defined by the presence of fibrosis as confirmed by Masson staining. All specimens were subjected to histological analysis with quantitative scoring. Detailed patient characteristics are provided in Supplementary Table 1. All studies involving human tissues were approved by the Institutional Review Board at the Second Affiliated Hospital of Nanjing Medical University. Informed consent was obtained from all patients for the use of their tissue samples in this research.

### Animals

All animals were maintained in the specific pathogen-free Laboratory Animal Center of Nanjing Medical University according to the guidelines of the Institutional Animal Care. This study was approved by the Ethics Committee of Nanjing Medical University (IACUC-2112054). Male C57BL/6 J mice (6-8 weeks old, 18-20 g) were sourced from the Specific Pathogen Free Laboratory Animal Center of Nanjing Medical University. Ksp-Cre transgenic mice (012237) on a C57BL/6 background were obtained from The Jackson Laboratory. Mice with a floxed Trim21 allele (exons 5-6 flanked by loxP sites) on a C57BL/6 background were acquired from Cyagen (SO201210DY1). To generate tubular cell-specific Trim21 knockout mice, Cre^+/-^, Trim21^fl/wt^ mice were first produced by crossing the floxed mice with Ksp-Cre transgenics. These heterozygous mice were then bred with homozygous Trim21^fl/fl^ mice to yield the knockout offspring (Trim21^fl/fl^, Cre^+/-^), herein referred to as Tub-Trim21^-/-^ (Supplementary Fig. S1). Cre-negative, homozygous Trim21^fl/fl^ littermates (genotype: Trim21^fl/fl^, Cre^-/-^) were used as control (herein referred to as Tub-Trim21^+/+^). The sample size was estimated based on previous experience and publications in similar animal models.

Unilateral ureteral obstruction (UUO) and ischemia-reperfusion injury (IRI) surgeries were performed as previously described [35]. Animals were sacrificed to perform analysis from 1–21 d after the surgery randomly with random number table. Renal tissues were harvested at designated time points post-surgery. Blinding was maintained during the experiment and outcome assessment.

### Trim21 shRNA delivery

A mouse Trim21-specific shRNA (target sequence: ACTCATGGCTCATCATCATCTCAA) was obtained from Integrated Biotech Solutions (Shanghai, China). To induce Trim21 knockdown, male C57BL/6 J mice received two tail vein injections of Trim21-shRNA (1 mg/kg per injection), administered three days before and three days after UUO surgery, respectively.

### Cell culture and treatment

Primary renal tubular cells were isolated from the kidneys of *Trim21*^fl/fl^ mice and cultured in Dulbecco’s Modified Eagle’s Medium-F12 (DMEM/F12; 12400024, Gibco) supplemented with 10% fetal bovine serum (FND500, ExCell Bio). To generate tubular cells with *Trim21* ablation, the cells were infected with an adenovirus carrying the Cre recombinase gene. For western blot analysis, primary renal tubular cells were pretreated with IFN-γ (SRP3058; Sigma-Aldrich) for 3 h followed by TGF-β1 (240-B-010-CF, R&D Systems) treatment for 48 h to induce ECM production. To inhibit protein degradation pathways, cells were treated with MG132 (M8699, Sigma-Aldrich) to inhibit the proteasome. *Loxl2*-specific and control small interfering RNAs (siRNAs; Integrated Biotech Solutions) were transiently transfected into tubular cells using Lipofectamine 3000 reagent (Invitrogen), according to the manufacturer’s instructions. Similarly, an HA-*Trim21* plasmid (TIANGEN BIOTECH) or the empty pCMV vector control (TIANGEN BIOTECH) was transiently transfected using Lipofectamine 3000. Following treatments, cells were collected for subsequent analyses.

### Histology and immunohistochemical staining

Mouse kidney samples were fixed in 10% neutral buffered formalin and embedded in paraffin. Subsequently, 3-μm sections were stained with periodic acid–Schiff (PAS) and Masson’s trichrome. Kidney biopsies from patients diagnosed with membranous nephropathy (MN), diabetic kidney disease (DKD), focal segmental glomerulosclerosis (FSGS), and IgA nephropathy (IgAN) were obtained from the Second Affiliated Hospital of Nanjing Medical University. For immunohistochemistry, slides were stained with anti-Trim21 antibody (12108-1-AP; 1:100 dilution). Renal injury was assessed based on the presence of tubular necrosis, cellular casts, and tubular damage using a semi-quantitative scoring system. The injury score was defined as follows: 0 ( < 10%), 1 (10–25%), 2 (25–50%), 3 (50–75%), and 4 ( > 75%) of the affected cortical area. For each mouse, at least ten randomly selected fields were evaluated under a light microscope (OLYMPUS DP74), and an average score was calculated.

### Real-time qRT-PCR assay

Total RNA was extracted with TRIzol reagent (Invitrogen) according to the manufacturer’s instructions. Complementary DNA (cDNA) was synthesized from 1 μg of total RNA using ReverTra Ace (Vazyme) and oligo(dT)₁₂–₁₈ primers. Quantitative real-time PCR (qRT-PCR) was performed on a LightCycler 96 system (Roche). The relative amount of mRNA to internal control was calculated using the equation 2 ΔCT, in which ΔCT = CT gene – CT control. The primer sequences used for RT-qPCR are listed in Supplementary Table S2.

### Western blot analysis

Kidney tissue homogenates and cell lysates were prepared using standard procedures, and Western blotting was performed as previously described. The following primary antibodies were used: anti-GAPDH (FL-335, Santa Cruz Biotechnology), anti-Loxl2 (BS90807, Bioworld), Anti-Trim21 (12108-1-AP, Proteintech), anti-Ubiquitin (sc-80017, Santa Cruz Biotechnology), anti-K48-ubiquitin (ab140601, abcam), K63-ubiquitin (ab179434, abcam), anti-Type I Collagen, (1310-01, Southern Biotech), anti-Fibronectin (F3648, Sigma-Aldrich), anti-Flag (F1804, Sigma-Aldrich), anti-HA (3724S, Cell Signal Technology), anti-Tubulin (sc-53646, Santa Cruz Biotechnology), anti-IFNG (BS3486, Bioworld), anti-IFNGR1 (BS78681, Bioworld), anti-STAT3 (4904S, Cell Signal Technology), anti-p-STAT3 (Y705) (9145S, Cell Signal Technology), anti-Smad3 (ab40854, abcam), anti-p-Smad1 (S463/465) /2 (S465/467) 3 (S423/425)/ 5 (S463/465) (ab52093, abcam).

### Mass spectrometry analysis

One-week-old Trim21 fl/fl mice without Cre were extracted primary renal tubular epithelial cells. These cells were treated with GFP or Cre-GFP virus for 36 h and were digested with trypsin and then washed three times with PBS. Cells (5×10^7^ cells) were harvested in an Eppendorf Safelock microcentrifuge tube. Protein extraction, enzymatic digestion, and TMT reagent labeling for identification and characterization were performed by BGI-GBI Biotech Co., Ltd. (Beijing, China). Peptides were separated by liquid chromatography, ionized using a nanoESI source, and analyzed with a Q-Exactive HF X mass spectrometer (Thermo Fisher Scientific) operating in Data-Dependent Acquisition (DDA) mode. Protein identification was performed using the NCBI search engine against the mouse RefSeq protein database. The upregulated proteins in Tub-Trim21^-/-^ cells compared to Tub-Trim21^+/+^ cells identified by proteomics are listed in Supplementary Table 3.

### Immunofluorescence staining

For cell staining, samples were fixed with 4% paraformaldehyde for 20 min, washed twice with PBS, permeabilized for 7 min, and blocked with 4% BSA for 45 min. For kidney cryosections, slides were fixed with 4% paraformaldehyde for 15 min at room temperature (RT) and permeabilized with 0.5% Triton X-100 for 10 min, followed by blocking with 10% donkey serum in 1×PBS for 1 h. Both cells and tissue sections were then incubated with primary antibodies overnight at 4 °C. After washing, samples were incubated with appropriate secondary antibodies for 2 h (cells) or 1 h (tissue) at RT in the dark. Nuclei were counterstained with DAPI (C0065, Solarbio). Slides were viewed using an OLYMPUS DP74 and BX53 epifluorescence microscope equipped with a digital camera.

### Immunoprecipitation

Primary tubular cells or HEK293A cells were washed three times with cold 1×PBS and lysed on ice for 10 min using IP lysis buffer (Beyotime) supplemented with 1% protease inhibitor cocktail and 1% phosphatase inhibitors I and II (Sigma-Aldrich). Lysates were centrifuged at 16,000 g for 15 min at 4 °C to collect the supernatant. The protein concentration was determined using a BCA assay kit (Pierce). For each immunoprecipitation, 500 µg of total protein was incubated with 1 µg of the target primary antibody overnight at 4 °C with gentle agitation. Protein A/G PLUS-Agarose beads (Santa Cruz) were then added, and the incubation continued for 2 h at 4 °C. The beads were subsequently washed three times with 1×PBS and bound proteins were eluted by boiling in 2×SDS sample loading buffer for subsequent immunoblotting analysis.

### Statistical analysis

All statistical analyses were performed using GraphPad Prism 8 software (GraphPad Software, San Diego, CA). Data are presented as mean ± SEM. Differences between two groups were assessed using a two-tailed unpaired Student’s t-test. For comparisons among more than two groups, one-way ANOVA followed by Dunnett’s post-hoc test was used. For data sets with two independent variables, two-way ANOVA followed by Tukey’s multiple comparisons test was applied. A *p*-value of less than 0.05 was considered statistically significant. Detailed statistical information for each experiment is provided in the corresponding figure legends.

## Supplementary information

Supplemental legends

Supplemental figure 1

Supplemental figure 2

Supplemental figure 3

Supplemental figure 4

Supplemental figure 5

Supplemental figure 6

Supplemental Table 1

Supplemental Table 2

Supplemental Table 3

WB original data

Reproducibility checklist

## Data Availability

The authors declare that all data supporting the findings of this study are available within the article and its Supplementary Information files, or from the corresponding author.
