# Supplementary material for: Pro-inflammatory cytokine IFN-γ protects against renal fibrosis by promoting E3 ubiquitin ligase Trim21-mediated Loxl2 degradation in tubular epithelial cells
Source: Cell Death Dis. 2026 May 13;17(1):619. doi: 10.1038/s41419-026-08850-7 (PMC13338432; doi:10.1038/s41419-026-08850-7)
Supplement: Supplementary file 1 — Supplemental legends [file 41419_2026_8850_MOESM1_ESM.docx]

**Supplementary Figure 1: Generating mouse model with tubular cell-specific deletion of Trim21.**

(a) Strategy for generating mice with tubular cell-specific deletion of Trim21. (b) Genotyping the mice with PCR analysis of genomic DNA as indicated. (c) Western blotting assay showing the reduction of Trim21 from kidney of Tub-Trim21^+/+^ and Tub-Trim21^-/-^. (d) Co-staining for Trim21 and tubular segment-specific markers PNA. Scale bar: 20 μm. The graphs showing Scr (e), BUN (f), NAG (g) and kidney/body weight index (h) between the knockouts and control littermates at 2 months after birth (n = 6). (i) PAS staining of kidney from Trim21 WT and KO mice at 12 months after birth (n=6). Blood SCr and urine NAG levels of Trim21 WT and KO mice at 2 or 12 months.

**Supplementary Figure 2: Knockdown of Trim21 with shRNA exacerbates UUO-induced renal fibrosis.**

(a) The construct strategy of Trim21 knockdown mice. (b) Western blotting analyses of Trim21 protein after injecting shRNA intravenously from kidney and liver of C57BL/6J mice. (c) Western blotting analyses of FN and α-SMA protein level from mice kidney of injecting Ctrl-shRNA and Trim21-shRNA plasmid after UUO surgery. (d) Quantitative analyses of FN and α-SMA from western blotting results with (c) (n=6). (e) Representative micrographs with PAS and Masson staining and FN immunofluorescence from mice kidney of injecting Ctrl-shRNA and Trim21-shRNA plasmid after UUO surgery. Bar: 20 μm. (f) Injury scores and the fibrotic area analyses from PAS and Masson staining in kidneys after UUO (n=6). Data are presented as the mean±SEM.**p*＜0.05, ***p*＜0.01, ****p*＜0.001, by two-way ANOVA with Tukey’s multiple-comparison test.

**Supplementary Figure 3:** **Neither overexpression nor ablation of Trim21 significantly altered the phosphorylation and nuclear translocation of Smad induced by TGF-β1.**

(a) The levels of p-Smad1/2/3/5 and total Smad3 proteins after overexpression of Trim21 and TGF-β1 treatment for 30 and 60 minutes. (b) Immunofluorescence images of Smad3 location in TECs treated with Trim21 overexpression and TGF-β1 for 60 minutes. Scale bar: 5 μm (c) The levels of p-Smad1/2/3/5 and total Smad3 proteins after ablation of Trim21 and TGF-β1 treatment for 30 and 60 minutes. (d) Immunofluorescence images of Smad3 location in TECs treated with Trim21 ablation and TGF-β1 for 60 minutes. Scale bar: 5 μm.

**Supplementary Figure 4: Verification of the knockdown efficiency of Loxl2 in TECs.**

(a-b) The mRNA and protein level of Loxl2 knockdown in primary TECs. Quantitative PCR and WB analysis were conducted in primary TECs at 24 h after Loxl2 siRNA transfection for 24 h. *****p* ＜ 0.05, by by two-tailed Student’s t test.

**Supplementary Figure 5: Knocking out Trim21 in the TECs had no effect on the transcriptional levels of the lox family genes.**

(a-e) The mRNA level of LOX family gene from Trim21^+/+^ or Trim21^-/-^ TECs. The TECs from Trim21 fl/fl mice were infected with adenovirus carrying GFP or GFP-Cre recombinase gene for 36 h, respectively.

**Supplementary Figure 6: Verification of expression of IFN-γin liver and kidney at 24 h after** **intravenous injection of plasmid.**

(a) Experimental design. Harvesting kidneys of C57BL/6J mice at 24 h after IFN-γ plasmid administration. (b-c) The mRNA levels of *Ifng* in livers and kidneys of C57BL/6J mice with or without IFN-γ plasmid (n=3). (d-e) The protein levels of IFN-γ in livers and kidneys of C57BL/6J mice with or without IFN-γ plasmid. (f) Representative micrographs with IFN-γ staining of livers and kidneys with or without IFN-γ plasmid treatment. Scale bar: 20 μm.

**Supplemental Table 1. Clinical Characteristics of the subjects**

**Supplemental Table 2.** **Primers used for Real Time-PCR (mouse)**

**Supplemental Table 3. Upregulated proteins in Tub-Trim21^-/-^ cells compared to Tub-Trim21^+/+^ cells identified by proteomics**
