# Supplementary figures and images for: Pro-inflammatory cytokine IFN-γ protects against renal fibrosis by promoting E3 ubiquitin ligase Trim21-mediated Loxl2 degradation in tubular epithelial cells
Source: Cell Death Dis. 2026 May 13;17(1):619. doi: 10.1038/s41419-026-08850-7 (PMC13338432; doi:10.1038/s41419-026-08850-7)

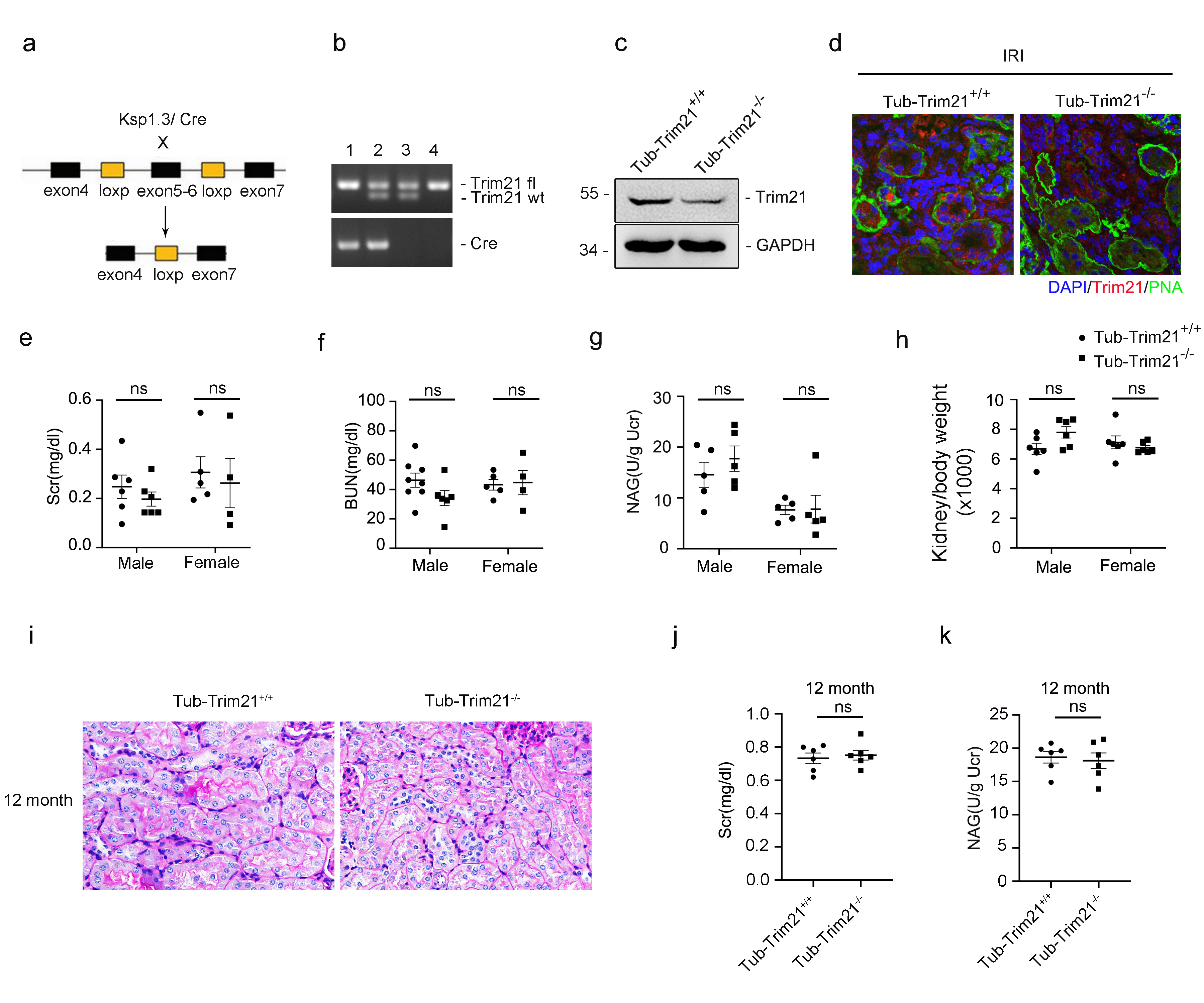

Supplement: Supplementary file 2 — Supplemental figure 1 [file 41419_2026_8850_MOESM2_ESM.tif]

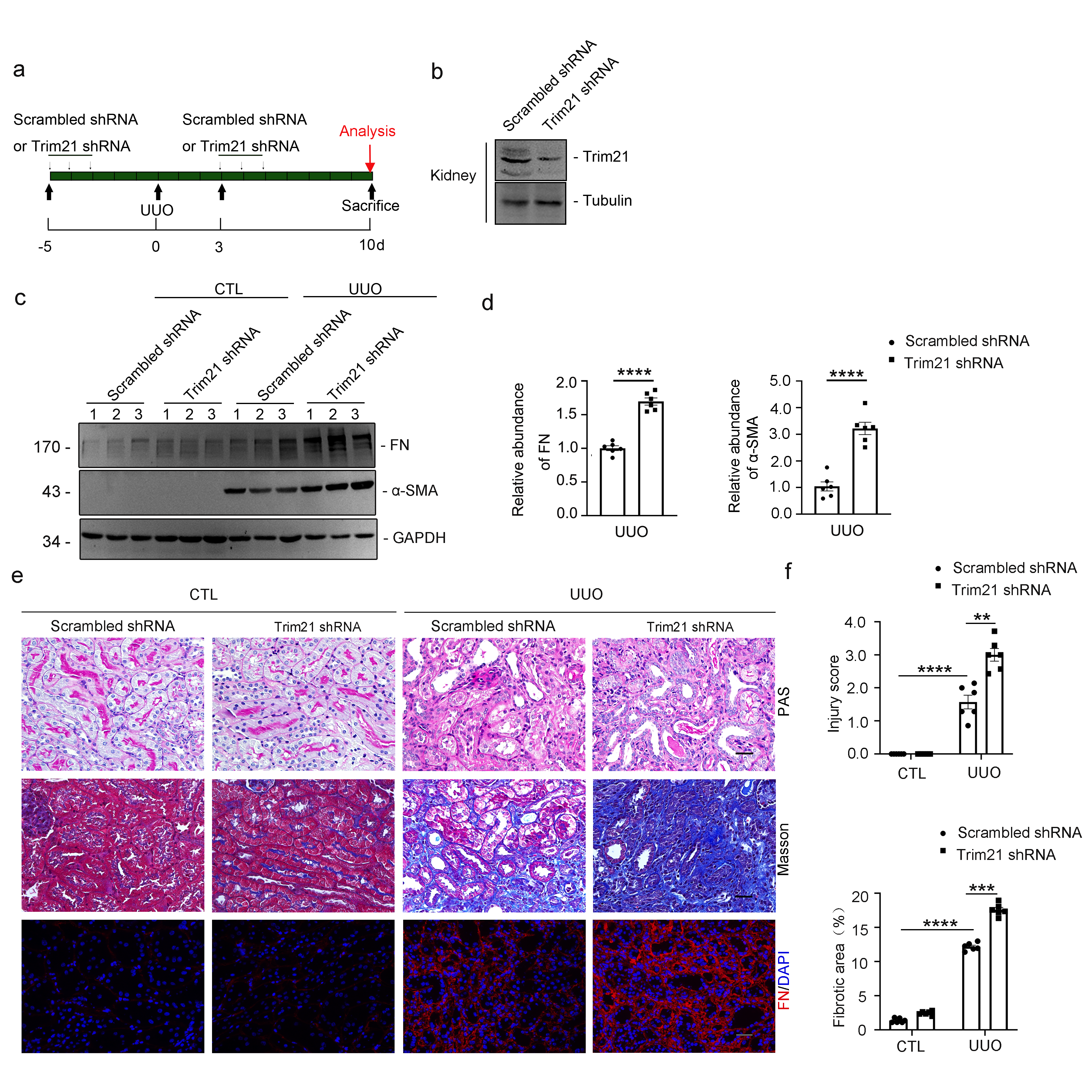

Supplement: Supplementary file 3 — Supplemental figure 2 [file 41419_2026_8850_MOESM3_ESM.tif]

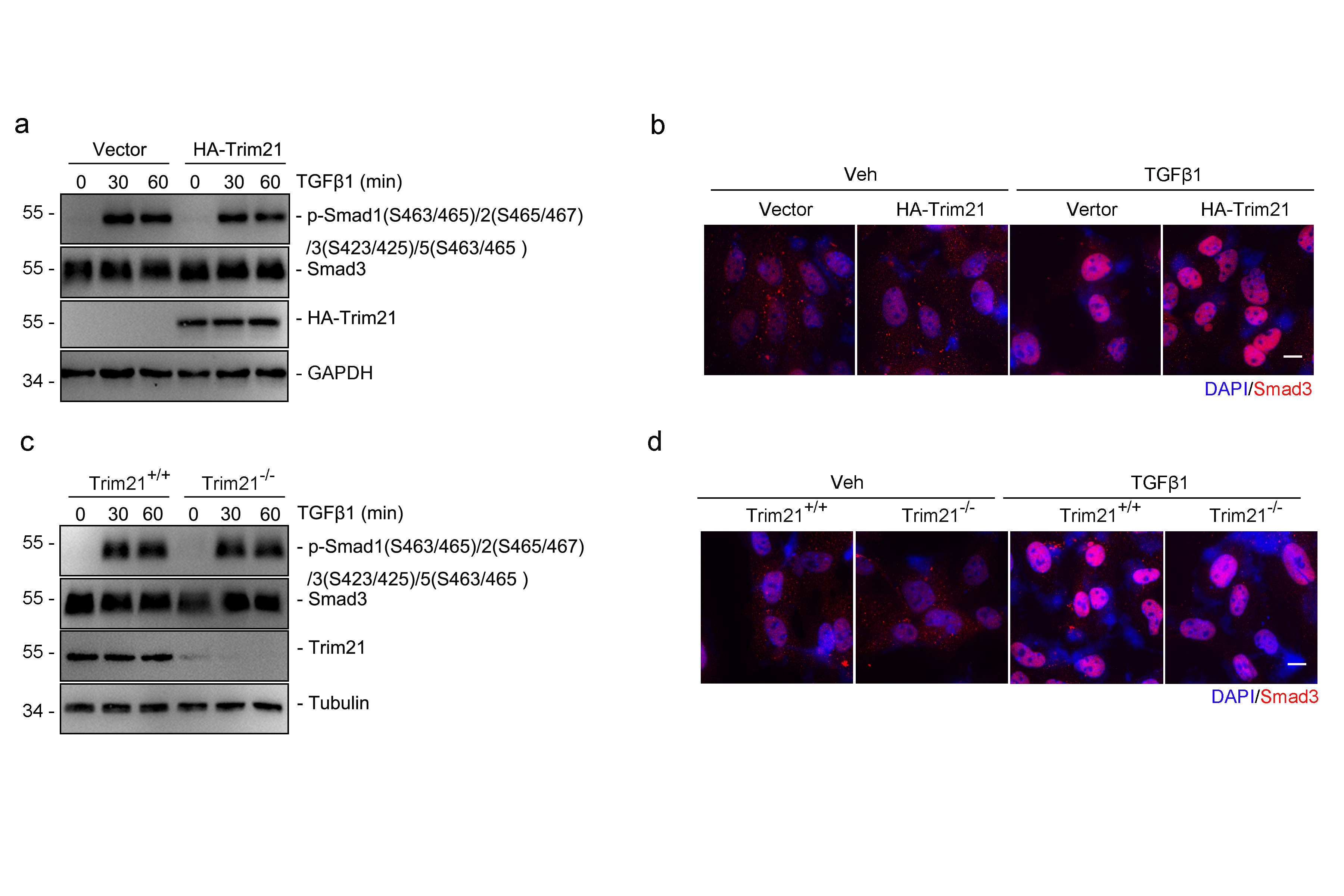

Supplement: Supplementary file 4 — Supplemental figure 3 [file 41419_2026_8850_MOESM4_ESM.tif]

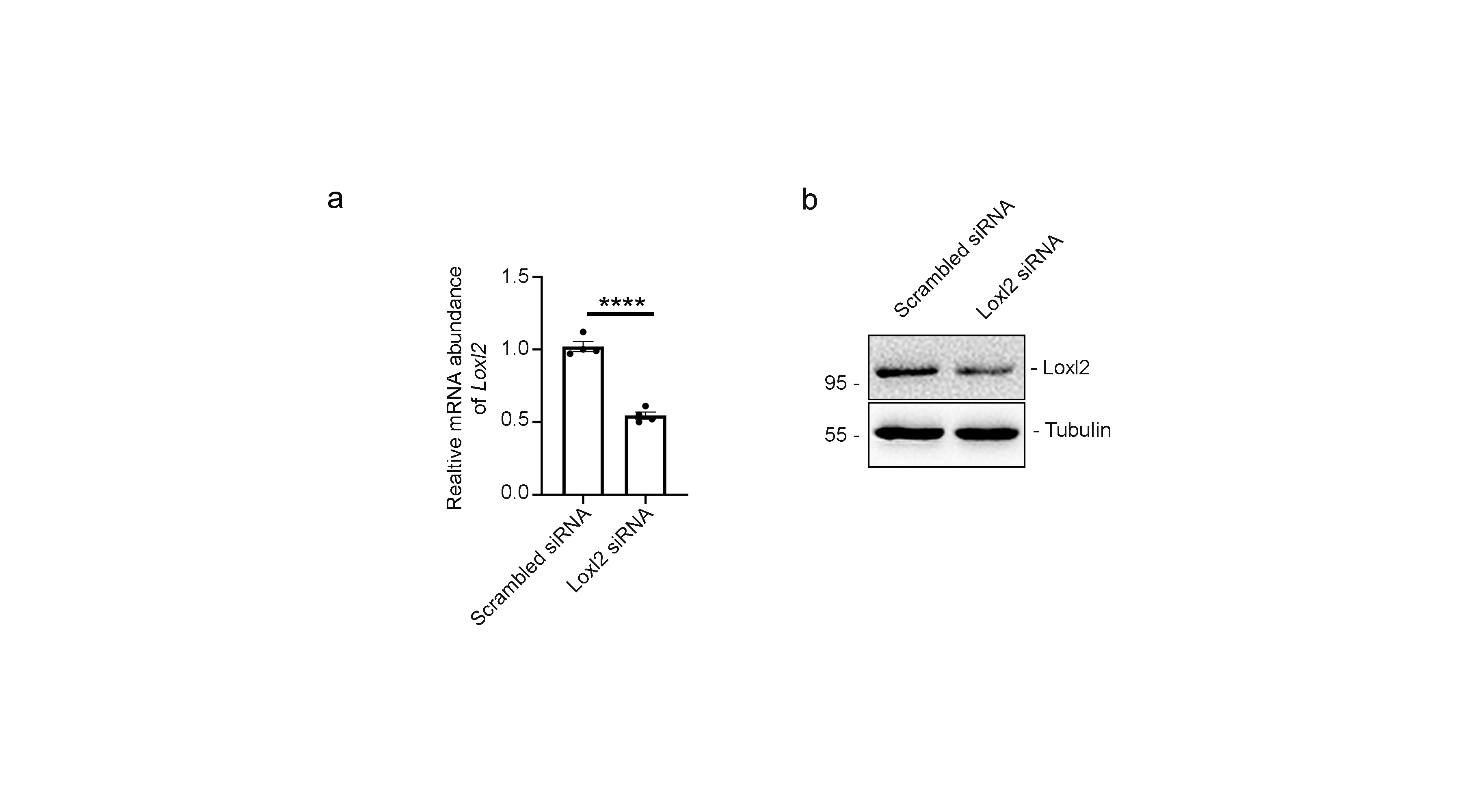

Supplement: Supplementary file 5 — Supplemental figure 4 [file 41419_2026_8850_MOESM5_ESM.tif]

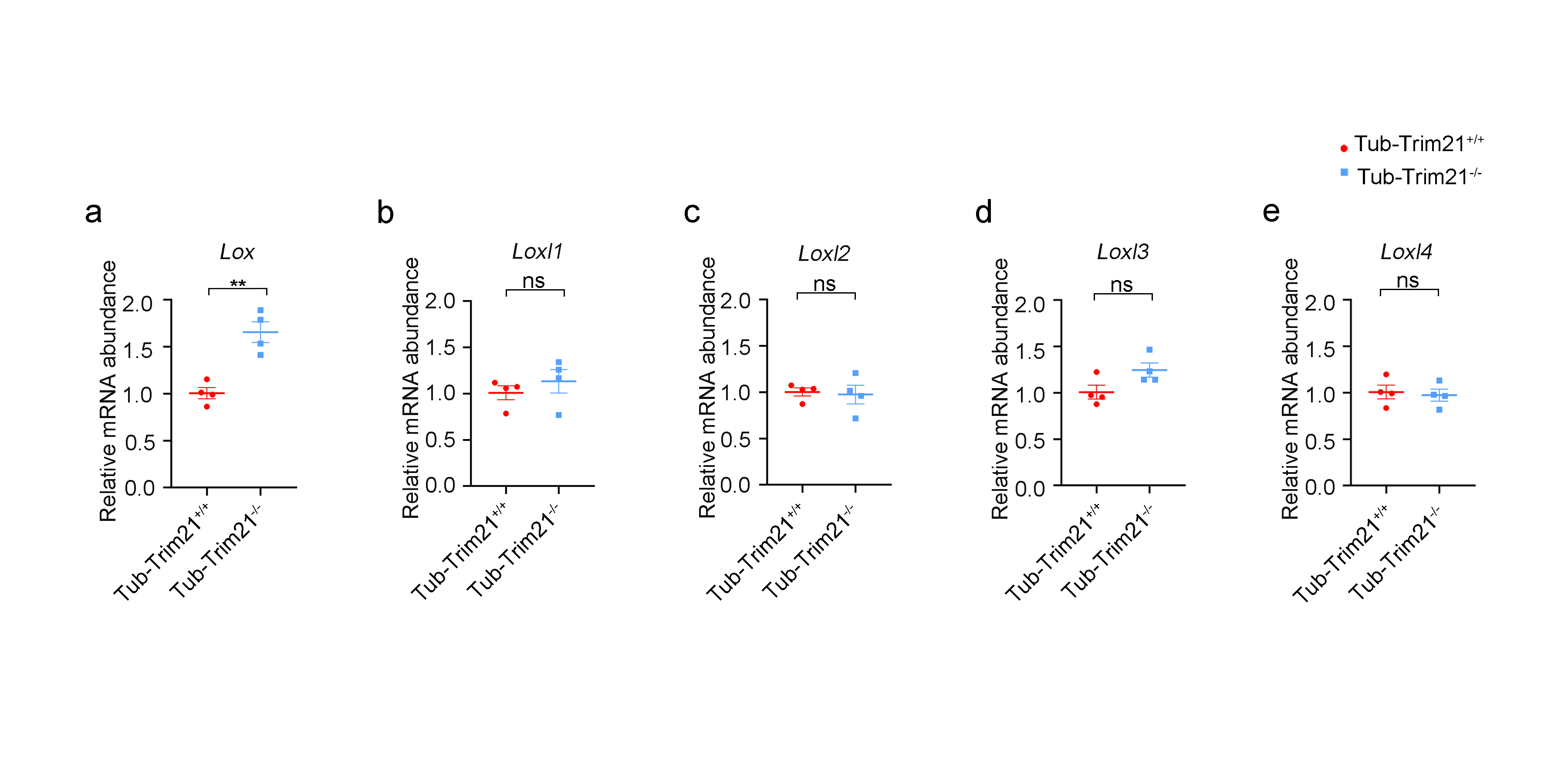

Supplement: Supplementary file 6 — Supplemental figure 5 [file 41419_2026_8850_MOESM6_ESM.tif]

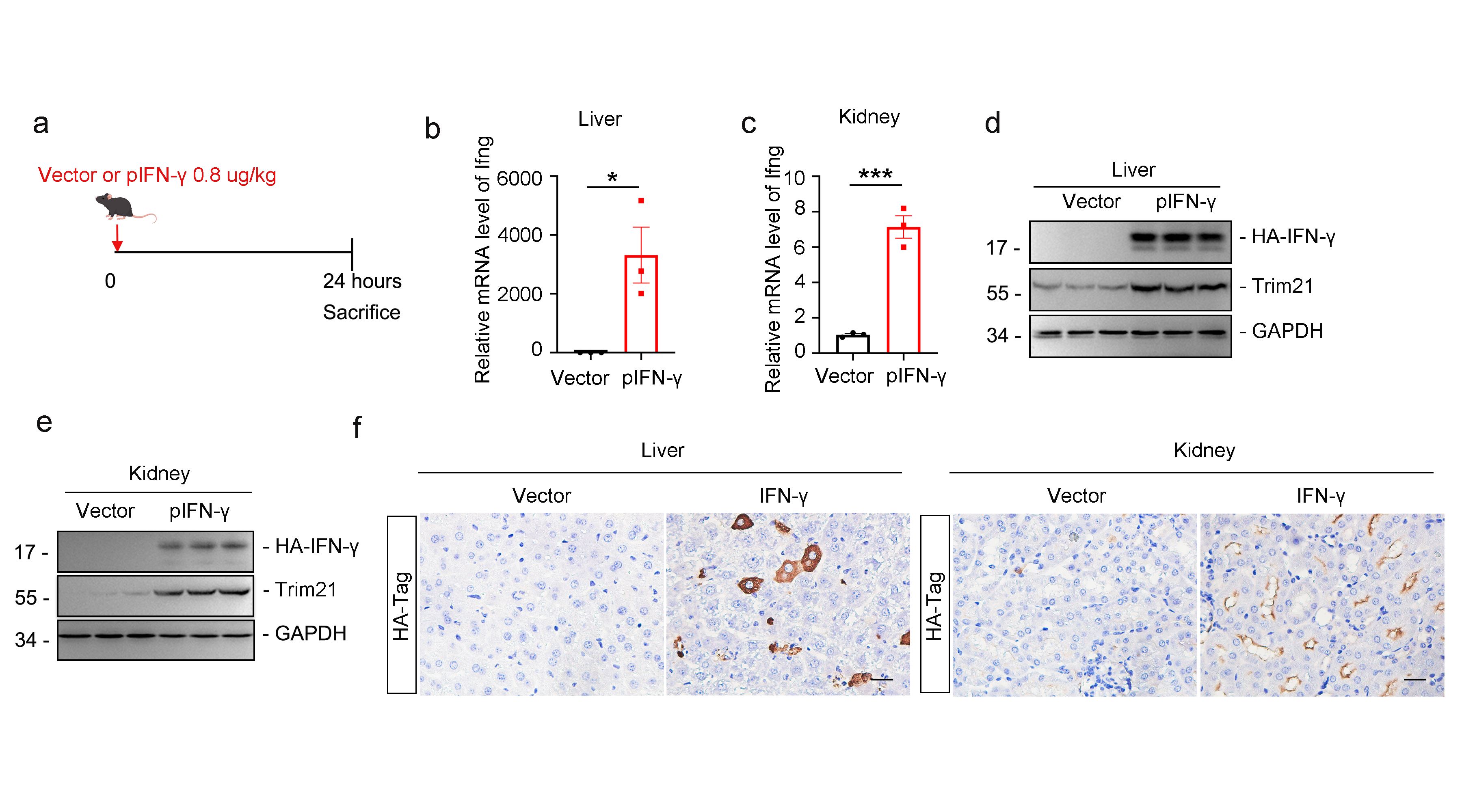

Supplement: Supplementary file 7 — Supplemental figure 6 [file 41419_2026_8850_MOESM7_ESM.tif]

Western blot original data

Figure 1d

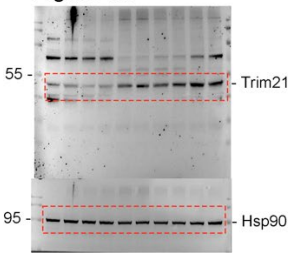

Figure 1g

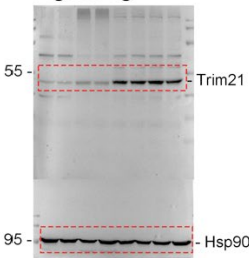

Figure 2a

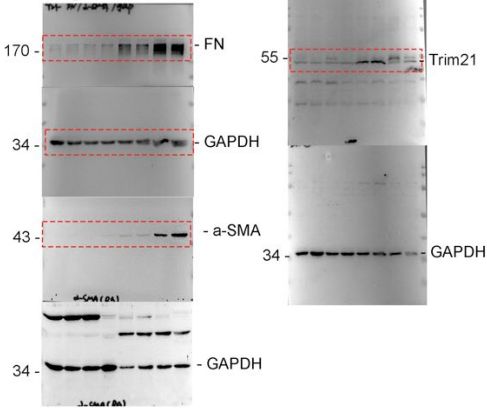

Figure 2e

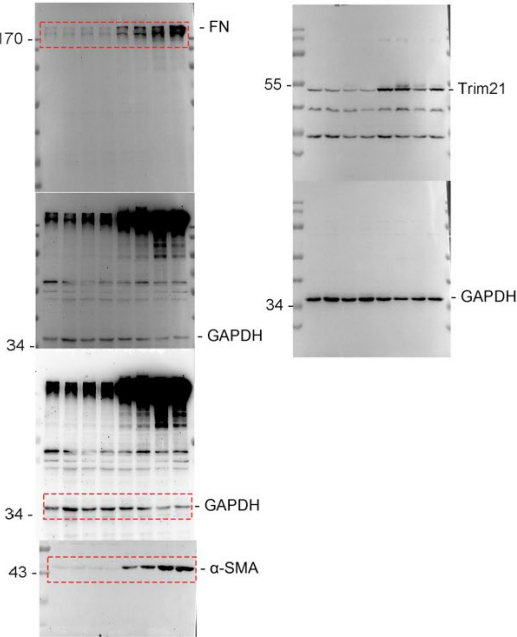

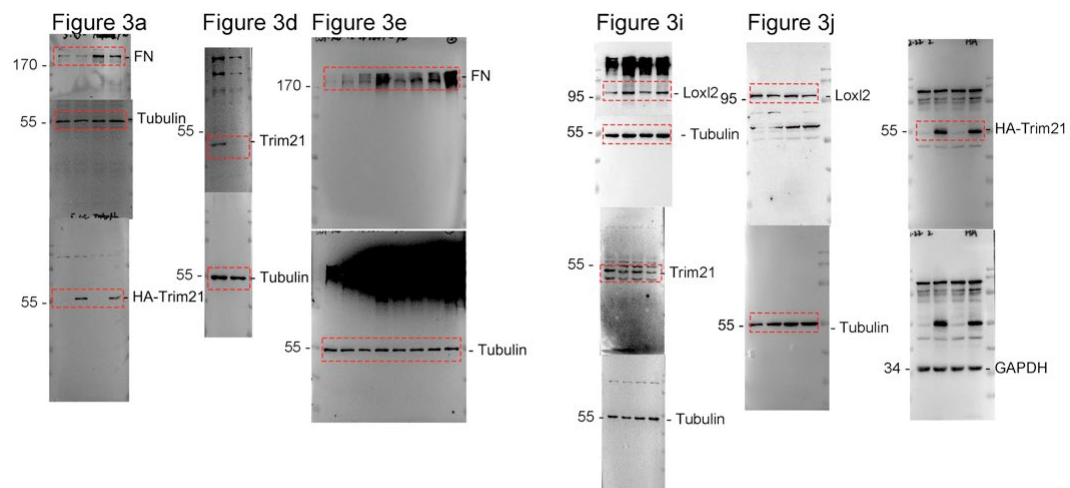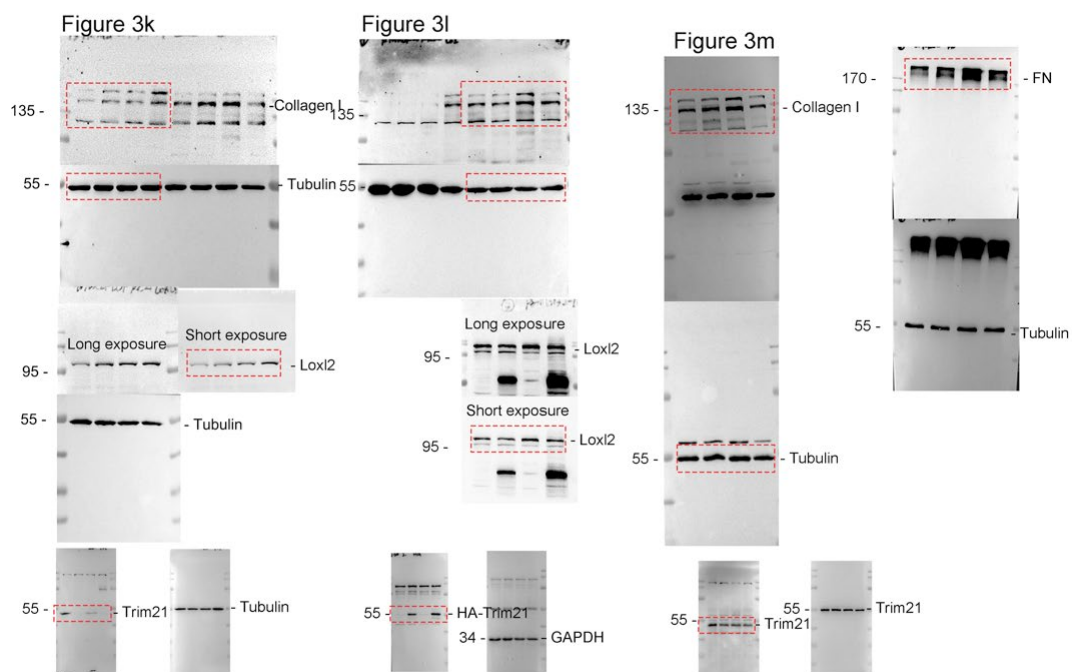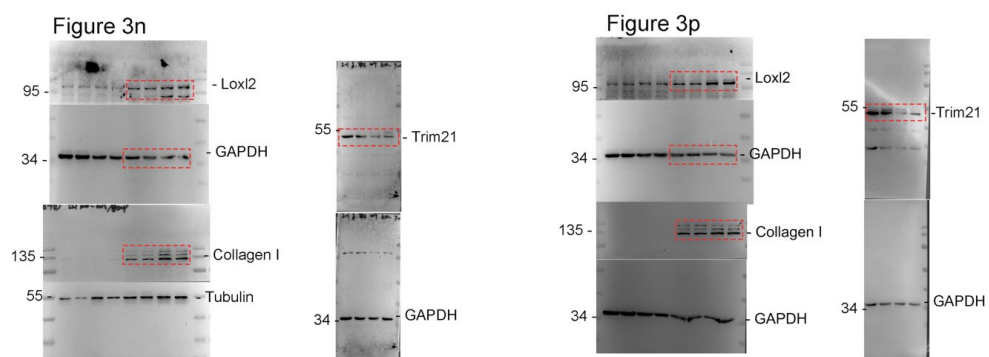

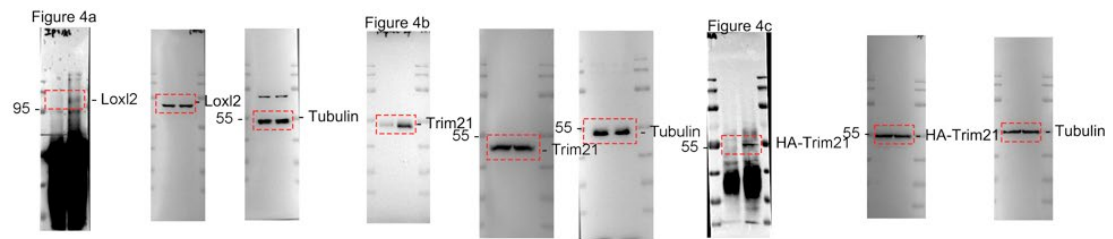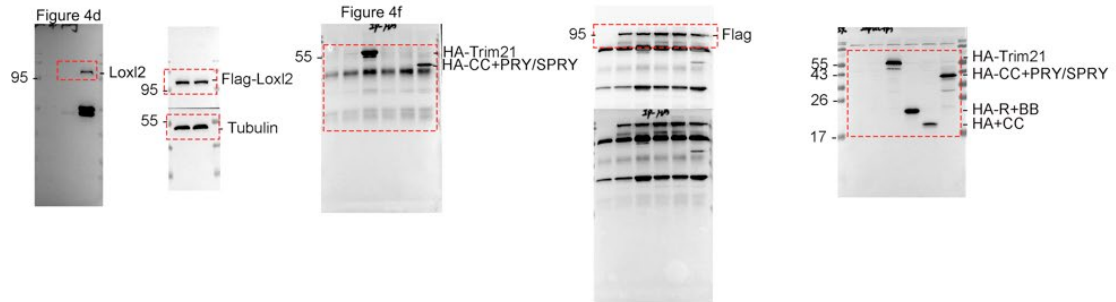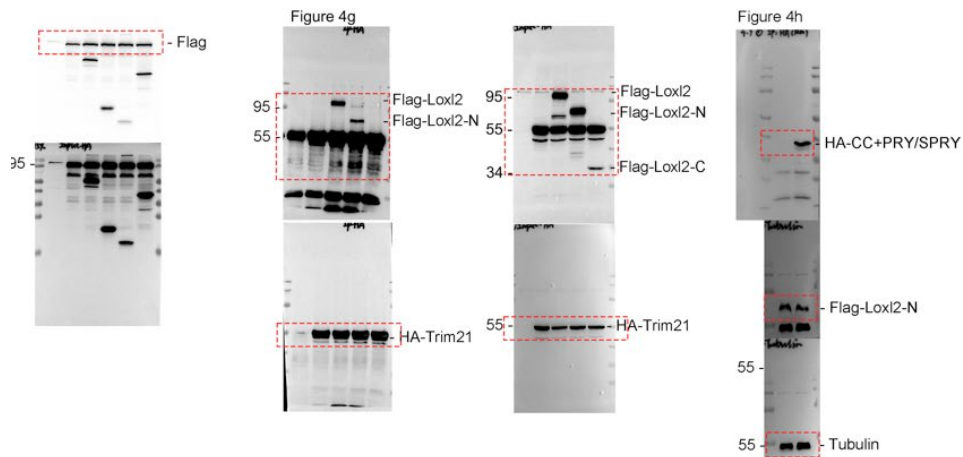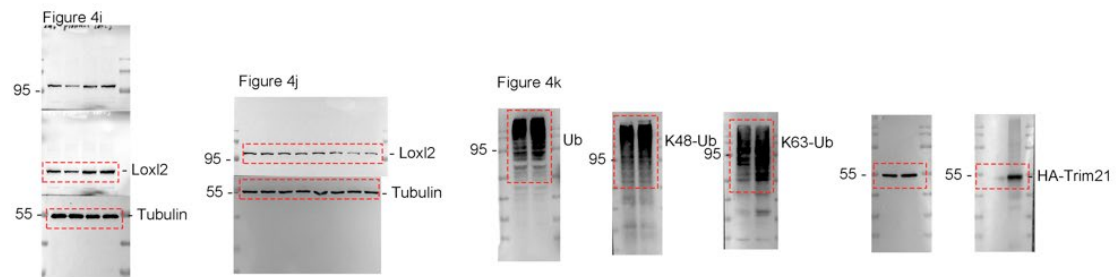

Figure 5m

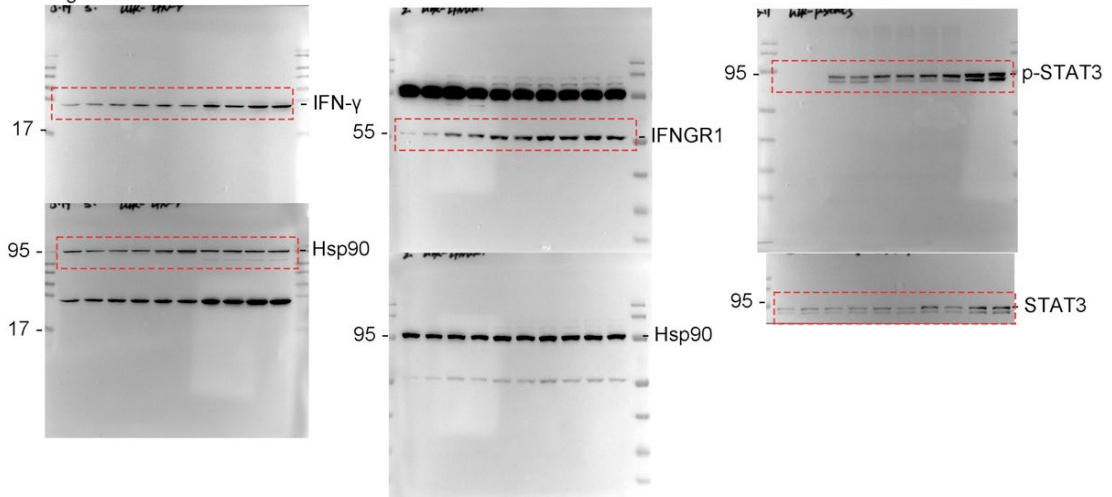

Figure 5n

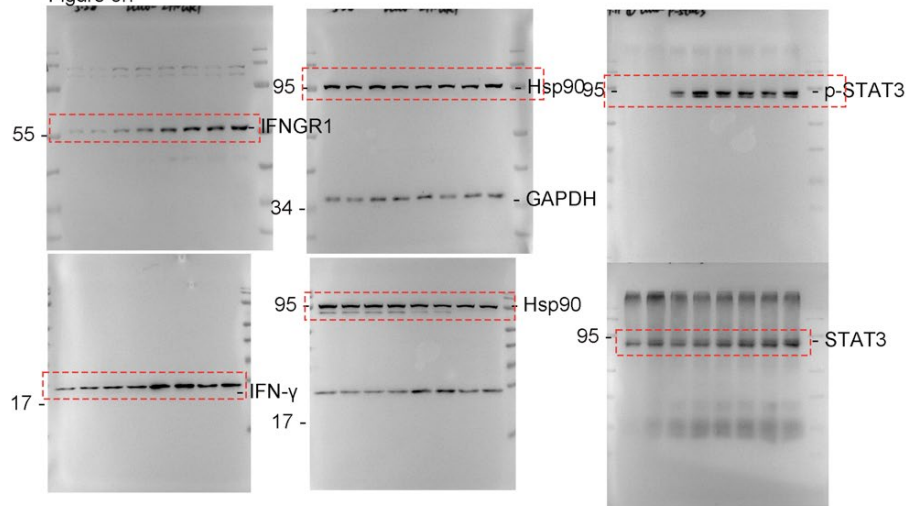

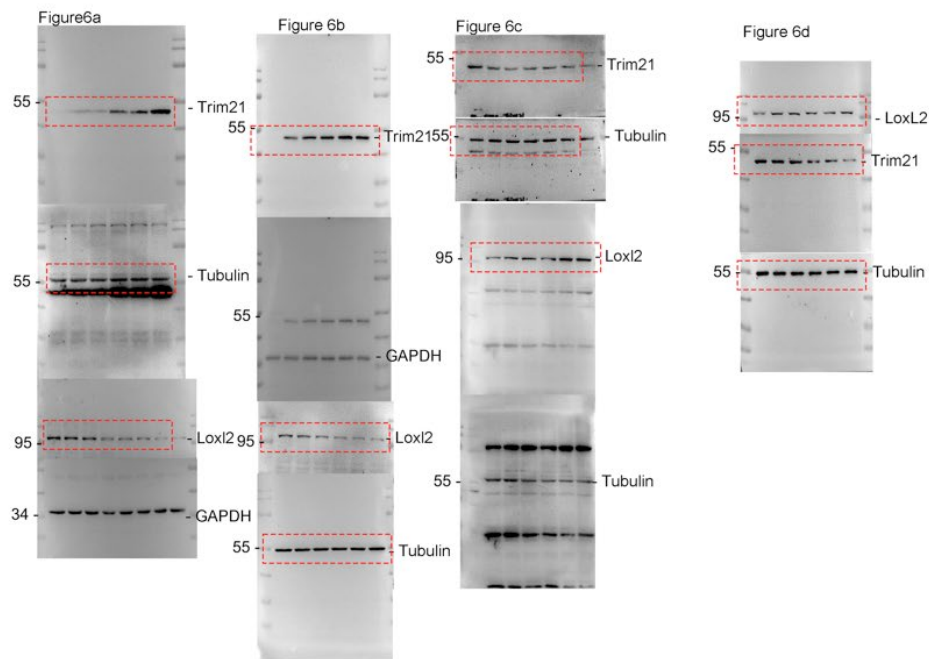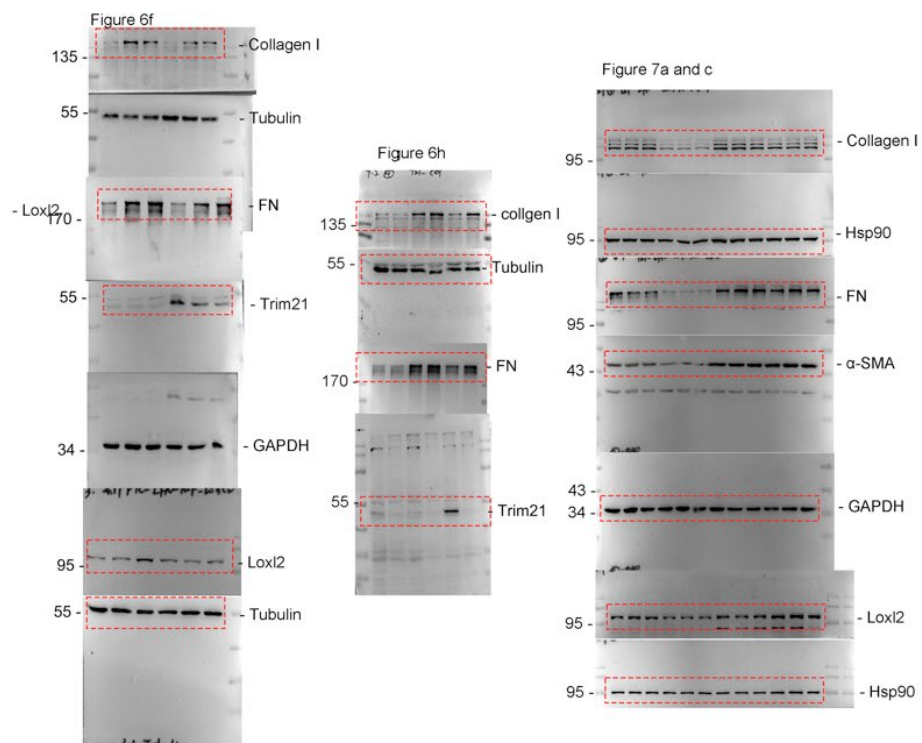

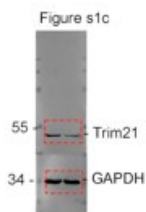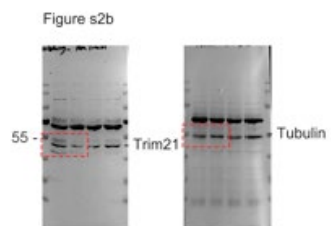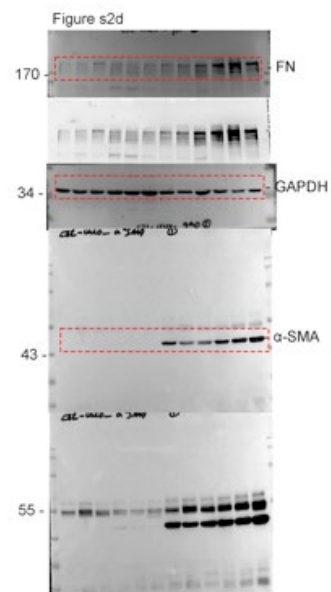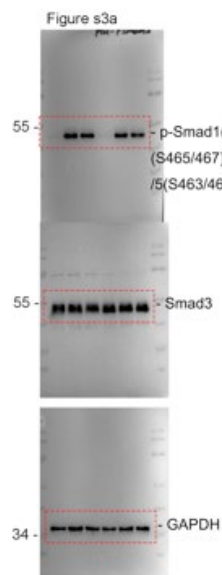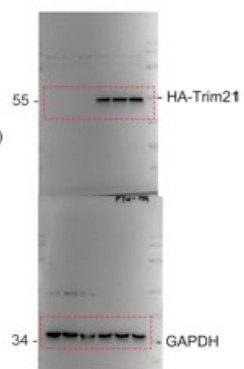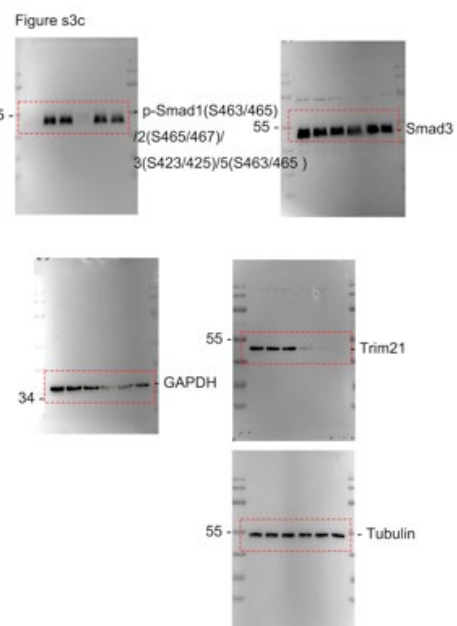

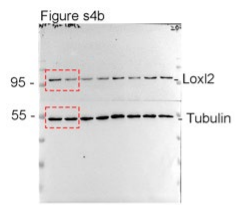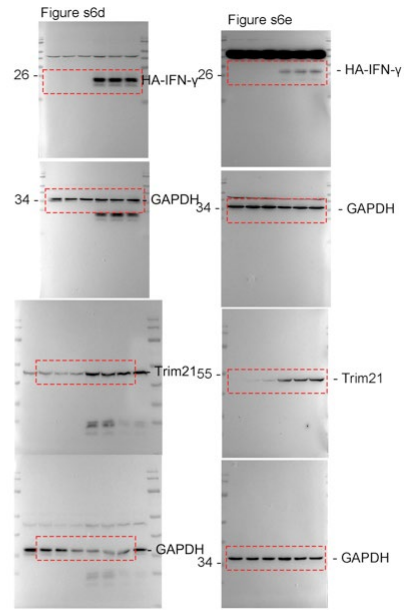

Supplement: Supplementary file 11 — WB original data [file 41419_2026_8850_MOESM11_ESM.pdf]
