# Supplementary material for: Pro-inflammatory cytokine IFN-γ protects against renal fibrosis by promoting E3 ubiquitin ligase Trim21-mediated Loxl2 degradation in tubular epithelial cells
Source: Cell Death Dis. 2026 May 13;17(1):619. doi: 10.1038/s41419-026-08850-7 (PMC13338432; doi:10.1038/s41419-026-08850-7)
Supplement: Supplementary file 8 — Supplemental Table 1 [file 41419_2026_8850_MOESM8_ESM.docx]

**Supplemental Table 1. Clinical Characteristics of the subjects**

|  | Normal Group  (n=8) | Subjects with renal fibrosis (n=20) |
| --- | --- | --- |
| Age (years) | 45.6+10.9 | 49.4+14.7 |
| Gender (male, n, %) | 3, (37.5%) | 7, (50%) |
| SCr (μmol/l) | 77.2+11.1 | 202.3+133.1 |
| BUN(mmol/l) | 6.1+1.1 | 11.8+7.1 |
| eGFR (ml/min/1.73m^2^) | 95.2+15.0 | 67.6+19.3 |
| MN (%) | - | 25% |
| DKD (%) | - | 25% |
| FSGS (%) | - | 25% |
| IgA (%) | - | 25% |
