# Supplementary material for: Pro-inflammatory cytokine IFN-γ protects against renal fibrosis by promoting E3 ubiquitin ligase Trim21-mediated Loxl2 degradation in tubular epithelial cells
Source: Cell Death Dis. 2026 May 13;17(1):619. doi: 10.1038/s41419-026-08850-7 (PMC13338432; doi:10.1038/s41419-026-08850-7)
Supplement: Supplementary file 9 — Supplemental Table 2 [file 41419_2026_8850_MOESM9_ESM.docx]

**Supplemental Table 2.** **Primers used for Real Time-PCR (mouse)**

| Gene name | Sequence (5’ to 3’) |
| --- | --- |
| *Trim21* | F: GGCACTCGGGACATGAACTG  R: GGGAGGAGGTCACCTGTTCTA |
| *Lox* | F: TCTTCTGCTGCGTGACAACC  R: GAGAAACCAGCTTGGAACCAG |
| *Loxl1* | F: GAGTGCTATTGCGCTTCCC  R: GGTTGCCGAAGTCACAGGT |
| *Loxl2* | F: AGTGGTATAGACAGGTCTGTTGG  R: ATTAACCCCAACTATGAAGTGCC |
| *Loxl3* | F: CTACTGCTGCTACACTGTCTGT  R: GACCTTCATAGGGCTTTCTAGGA |
| *Loxl4* | F: CATTATTCCAGTAGTCGGGGATG  R: AGCCACAGCACTCAAACAG |
| *Ifng* | F: CCATCCTTTTGCCAGTTCCTC  R: ATGAACGCTACACACTGCATC |
| *Ifngr1* | F: GCATACGACAGGGTTCAAGTTAT  R: CTGGCAGGATGATTCTGCTGG |
| *Ifngr2* | F: GTCTTGGGTCATTGCTGGAAG  R: TCCTCGCCAGACTCGTTTTC |
| *Fn1* | F: TTCAAGTGTGATCCCCATGAAG  R: CAGGTCTACGGCAGTTGTCA |
| *Acta2* | F: CCCAGACATCAGGGAGTAATGG  R: TCTATCGGATACTTCAGCGTCA |
| *Gapdh* | R: CGGCTCTAATCATAGTTGGGTCT  F: TGTAGACCATGTAGTTGAGGTCA |
| *Actin* | R: GGCTGTATTCCCCTCCATCG  F: CCAGTTGGTAACAATGCCATGT |
